# Supplementary material for: Dynamics of sociopolitical polarization and effects of misperception-correcting information around the 2022 Brazilian elections
Source: Nat Commun. 2026 Jun 5;17:4862. doi: 10.1038/s41467-026-72990-9 (PMC13241496; doi:10.1038/s41467-026-72990-9)
Supplement: Supplementary file 1 — Supplementary Information [file 41467_2026_72990_MOESM1_ESM.pdf]

Supplementary Materials

Supplementary Tables 4

Supplementary Table 1 | Randomized treatment conditions 4

Supplementary Table 2 | Socio-demographic characteristics over waves 8

Supplementary Table 3 | Treatment conditions of participants over repeated waves 9

Supplementary Table 4 | Preregistered results of affective polarization over waves 14

Supplementary Table 5a | Bayes Factors in the direction of the alternative hypothesis (BF_{10}) and equivalence tests (changes in affective polarization throughout the 2022 election considering the vote intention group definition) 15

Supplementary Table 5b | Bayes Factors in the direction of the alternative hypothesis (BF_{10}) and equivalence tests (changes in affective polarization throughout the 2022 election considering the ideological left-right self-identification and anti-politician group definitions) 16

Supplementary Table 5c | Bayes Factors in the direction of the alternative hypothesis (BF_{10}) and equivalence tests (changes in affective meta-polarization throughout the 2022 election considering the vote intention group definition) 17

Supplementary Table 5d | Bayes Factors in the direction of the alternative hypothesis (BF_{10}) and equivalence tests (changes in affective polarization throughout the 2022 FIFA World Cup considering the vote intention group definition) 18

Supplementary Table 5e | Bayes Factors in the direction of the alternative hypothesis (BF_{10}) and equivalence tests (treatment effects of misperception-correcting information relating to abortion) 19

Supplementary Table 5f | Bayes Factors in the direction of the alternative hypothesis (BF_{10}) and equivalence tests (treatment effects of misperception-correcting information relating to Amazonian deforestation) 20

Supplementary Table 5g | Bayes Factors in the direction of the alternative hypothesis (BF_{10}) and equivalence tests (treatment effects of misperception-correcting information relating to social quotas) 21

Supplementary Table 5h | Bayes Factors in the direction of the alternative hypothesis (BF_{10}) and equivalence tests (treatment effects of misperception-correcting information relating to racial quotas) 22

Supplementary Table 5i | Bayes Factors in the direction of the alternative hypothesis (BF_{10}) and equivalence tests (treatment effects of misperception-correcting information on liking scores across waves) 23

Supplementary Table 5j | Bayes Factors in the direction of the alternative hypothesis (BF_{10}) and equivalence tests (treatment effects of misperception-correcting information on perceived intelligence scores across waves) 24

Supplementary Table 5k | Bayes Factors in the direction of the alternative hypothesis (BF_{10}) and equivalence tests (treatment effects of misperception-correcting information on policy support for abortion and Amazonian deforestation) 25

Supplementary Table 5l | Bayes Factors in the direction of the alternative hypothesis (BF_{10}) and equivalence tests (treatment effects of misperception-correcting information on policy support for social and racial quotas) 26

Supplementary Table 5m | Bayes Factors in the direction of the alternative hypothesis (BF_{10}) and equivalence tests (changes in political identity salience throughout the 2022 election) 27

Supplementary Table 6 | Actual and perceived policy support among bolsonaristas and lulistas 28

Supplementary Table 7 | Effects of misperception-correcting information on liking and perceived intelligence scores 29

Supplementary Table 8 | Effects of misperception-correcting information on policy support 30

Supplementary Table 9 | Benjamin-Hochberg correction for multiple hypotheses testing . 31

Supplementary Figures 32

Supplementary Fig. 1 | Co-occurrence of political affiliation considering alternative grouping criteria. 32

Supplementary Fig. 2 | Evolution of affective polarization over waves considering preregistered models, using first-round election intention-to-vote definition of pro-person groups (lulistas and bolsonaristas). 33

Supplementary Fig. 3 | Evolution of affective polarization using anti-Lula and anti-Bolsonaro sentiment 34

Supplementary Fig. 4 | Sensitivity analyses showing evolution of affective polarization for lulistas and bolsonaristas with replacement of missing values by values along the interval [-.20 SD, + .20 SD] of the respondents' liking and perceived intelligence scores reported in the last wave they had provided such scores. 35

Supplementary Fig. 5 | Evolution of affective polarization for lulistas and bolsonaristas over waves considering only participants assigned to control conditions. 37

Supplementary Fig. 6 | Election effects across different model specifications. 38

Supplementary Fig. 7 | Evolution of affective polarization for lulistas' and bolsonaristas' evaluations of ingroups and outgroups, using the intention-to-vote definition. 39

Supplementary Fig. 8 | Evolution of affective polarization for lulistas' and anti-lulistas' evaluations of ingroups and outgroups. 40

Supplementary Fig. 9 | Evolution of affective polarization for bolsonaristas' and anti-bolsonaristas' evaluations of ingroups and outgroups. 41

Supplementary Fig. 10 | Evolution of affective polarization for self-identified left-wing and right-wing considering evaluated targets that talk little and a lot about politics. 42

Supplementary Fig. 11 | Evolution of meta-polarization over waves. 43
Supplementary Fig. 12 | Evolution of policy support. 44
Supplementary Fig. 13 | Evolution of affective polarization during the World Cup. 45
Post-election events: 47
Supplementary Note 1: The World Cup and storming of federal government buildings 47
Supplementary Fig. 14 | Effect of Brazil team's performance on affective polarization during the World Cup. 49
Supplementary Fig. 15 | Evolution of affective polarization during the World Cup considering self-identified left-wing and right-wing respondents. 50
Supplementary Fig. 16 | Evolution of affective polarization during the World Cup considering anti-Lula and anti-Bolsonaro respondents 51
Supplementary Fig. 17 | Treatment effects across different model specifications. 52
Supplementary Fig. 18 | The effect of providing misperception-correcting information for the ingroups and outgroups of lulistas and bolsonaristas in waves 2 and 4. 53
Supplementary Fig. 19 | The effect of misperception-correcting information for the ingroups and outgroups of lulistas and bolsonaristas in waves 3 and 5 54
Supplementary Fig. 20 | The effect of providing misperception-correcting information on liking and perceived intelligence scores for the ingroups and outgroups of self-identified left-wing and right-wing individuals. 55
Supplementary Fig. 21 | The effect of providing misperception-correcting information on liking and perceived intelligence scores for the ingroups and outgroups of people holding anti-Lula or anti-Bolsonaro sentiment. 56
Supplementary Fig. 22 | The effect of providing misperception-correcting information on policy support for self-identified left-wing and right-wing individuals. 57
Supplementary Fig. 23 | The effect of providing misperception-correcting information on policy support for people holding anti-Lula or anti-Bolsonaro sentiment. 58
Supplementary Fig. 24 | The effect of providing misperception-correcting information on policy support by wave. 59
Supplementary Fig. 25 | Election and treatment effects 60
Supplementary Fig. 26 | Percentage of participants who reported that their political identity is one of the three most important aspects of their sense of identity in wave 3 61
Supplementary Fig. 27 | Comparing the salience of political group definitions 62
Supplementary Fig. 28 | Evolution of issue polarization for lulistas and bolsonaristas. 63
Supplementary Fig. 29 | Google trends on abortion, deforestation, racial and social quotas in Brazil from Jan 2021 to Feb 2022 64
References 65

Supplementary Tables

Supplementary Table 1 | Randomized treatment conditions

Abortion Condition:

Currently, abortion is permitted in Brazil only in three situations: when there is a risk to the woman's life, when the pregnancy is a result of rape, or when the fetus is anencephalic (has not developed part or all of the brain). In all other cases, abortion is prohibited in Brazil.

However, there is significant debate surrounding abortion. Some people advocate for relaxing the rules to allow abortion for any reason, but under the guidance of professionals and provided it is done within the first three months of pregnancy. Others oppose this proposal, often arguing for even stricter regulations on abortion.

Now, think about bolsonaristas, those who intend to vote for Jair Bolsonaro in the first round of this year's elections. Out of every 10 bolsonaristas, how many do you believe support the proposal to relax abortion regulations to allow it up to the 3rd month of pregnancy? __

Now, think about lulistas, those who intend to vote for Lula in the first round of this year's elections. Out of every 10 lulistas, how many do you believe support the proposal to relax abortion regulations to allow it up to the 3rd month of pregnancy? __

In an earlier round of this study, we asked Lula supporters and Bolsonaro supporters their opinions on the decriminalization of abortion up to the 3rd month of pregnancy.

Very few Bolsonaro supporters, only 11%, said they were in favor of decriminalizing abortion up to the 3rd month of pregnancy. The percentage of supporters was slightly higher among Lula supporters. However, even so, less than half (only 46%) of Lula supporters indicated that they were in favor of decriminalizing abortion.

Amazon Zero Deforestation Condition:

Currently, deforestation of native vegetation in the Amazon for alternative land use (e.g., agriculture, livestock) is permitted in Brazil as long as certain requirements are met. For example, compensatory measures must be adopted to ensure the conservation of endangered species, and authorization must be obtained from the relevant state agency.

However, there is significant debate surrounding deforestation in the Amazon. Some people advocate for further relaxing the rules to allow deforestation to some extent in order to economically develop the region, albeit with restrictions. Others oppose this proposal, advocating for stricter controls to prevent any level of deforestation in the Amazon, aiming for Zero Deforestation.

Now, think about bolsonaristas, those who intend to vote for Jair Bolsonaro in the first round of this year's elections. Out of every 10 bolsonaristas, how many do you believe support the Zero Deforestation proposal for the Amazon? __

Now, think about lulistas, those who intend to vote for Lula in the first round of this year's elections. Out of every 10 lulistas, how many do you believe support the Zero Deforestation proposal for the Amazon? __

In an earlier round of this study, we asked Lula supporters and Bolsonaro supporters their opinions on zero deforestation.

The majority of Lula supporters, 80%, said they were in favor of zero deforestation in the Amazon. The percentage of supporters was slightly lower among Bolsonaro supporters. Nevertheless, even so, the majority of Bolsonaro supporters (63%) indicated that they were in favor of zero deforestation in the Amazon.

Social Quotas Condition:

Today, 50% of the spots offered at public universities are reserved for students who attended public schools. Of this quota, half are allocated to students from public schools with a family income equal to or less than one and a half minimum wage per capita and the other half to students from public schools with a family income above one and a half minimum wage per capita. Public school students must go through the selection process, either through the ENEM or entrance exams, and are classified within the quota spots.

However, there is significant debate surrounding quotas for public school students at universities. Some people argue that these quotas are unfair and should be abolished, with university access granted solely based on individual merit. Others, on the other hand, advocate for expanding income-based quotas at public universities.In an earlier round of this study, we asked Lula supporters and Bolsonaro supporters their opinions on quotas for low-income families in public universities.

Now, think about bolsonaristas, those who voted for Jair Bolsonaro in the first round of this year's elections. Out of every 10 bolsonaristas, how many do you believe support quotas for people from low-income families at universities? __

Now, think about lulistas, those who voted for Lula in the first round of this year's elections. Out of every 10 lulistas, how many do you believe support quotas for people from low-income families at universities? __

In an earlier round of this study, we asked Lula supporters and Bolsonaro supporters their opinions on quotas for low-income families in public universities.

The majority of Lula supporters, 93%, said they were not against quotas for low-income families in public universities. The percentage of those who were not opposed to the policy was slightly lower among Bolsonaro supporters. Nevertheless, the majority of Bolsonaro supporters (79%) indicated that they were not against quotas for low-income families in public universities.

Racial Quotas:

Today, 50% of the spots offered at public universities are reserved for students who attended public schools. Of these, half are allocated to students from public schools with a gross family income equal to or less than one and a half minimum wages per capita, and the other half to students from public schools with a family income above one and a half minimum wages. Additionally, within each income category, there are racial quotas, with spots reserved for Afro-descendants (Black and Brown) and Indigenous people. Black, Brown, and Indigenous students from public schools must go through the selection process, either through the ENEM or entrance exams, and are classified within the quota spots.

However, there is significant debate around racial quotas. Some people argue that they are unfair and should be abolished, with university access granted solely based on individual merit. Others, on the other hand, advocate for expanding racial quotas at public universities.

Now, think about bolsonaristas, those who voted for Jair Bolsonaro in the first round of this year's elections. Out of every 10 bolsonaristas, how many do you believe support quotas for Afro-descendants at public universities? __

Now, think about lulistas, those who voted for Lula in the first round of this year's elections. Out of every 10 lulistas, how many do you believe support quotas for Afro-descendants at public universities? __

In an earlier round of this study, we asked Lula supporters and Bolsonaro supporters their opinions on quotas for Afro-descendants in public universities.

The majority of Lula supporters, 87%, said they were not against quotas for Afro-descendants in public universities. The percentage of those who were not opposed to the policy was slightly lower among Bolsonaro supporters. Nevertheless, nearly half of Bolsonaro supporters (46%) indicated that they were not against quotas for Afro-descendants in public universities.

Supplementary Table 2 | Socio-demographic characteristics over waves

	Wave 1	Wave 2	Wave 3	Wave 4	Wave 5
Age (M)	40.03	39.50	40.00	40.87	41.14
Female (%)	49.21	50.73	49.91	49.25	49.31
Education (%)					
High School Not Completed or Lower	72.56	73.52	71.31	70.82	68.35
High School Completed or Over	27.44	26.48	28.69	29.18	30.65
Income (%)					
Less than 3 MW	61.06	59.99	58.46	55.39	52.79
More than 3 MW	38.94	40.01	41.54	44.61	47.21
Region					
Northeast	28.03	28.22	27.61	27.61	27.54
Southeast	40.05	37.50	38.72	39.09	38.73
South	14.68	17.64	18.24	17.58	17.91
Midwest	11.87	11.16	10.07	10.86	10.43
North	5.37	5.49	5.36	4.86	5.38

Supplementary Table 3 | Treatment conditions of participants over repeated waves

Wave 2	Wave 3	Wave 4	Wave 5	Frequency
Amazon	Abortion	Social quotas	Racial quotas	38
Amazon	Abortion	Racial quotas	Social quotas	53
Amazon	Abortion	Control	Control	107
Amazon	Abortion	.	Social quotas	11
Amazon	Abortion	.	Racial quotas	5
Amazon	Abortion	.	Control	17
Amazon	Abortion	Social quotas	.	4
Amazon	Abortion	Racial quotas	.	2
Amazon	Abortion	Control	.	8
Amazon	Abortion	.	.	30
Amazon	Abortion	Racial quotas	Control	1
Amazon	Control	Social quotas	Racial quotas	36
Amazon	Control	Racial quotas	Social quotas	53
Amazon	Control	Control	Control	96
Amazon	Control	.	Social quotas	14
Amazon	Control	.	Racial quotas	12
Amazon	Control	.	Control	21
Amazon	Control	Social quotas	.	6
Amazon	Control	Racial quotas	.	7
Amazon	Control	Control	.	12
Amazon	Control	.	.	24
Amazon	Control	Social quotas	Social quotas	1
Amazon	Control	Racial quotas	Control	1
Amazon	Control	Control	Social quotas	2

Amazon	.	.	.	109
Amazon	.	Social quotas	Racial quotas	2
Amazon	.	Racial quotas	Social quotas	5
Amazon	.	Control	Control	18
Amazon	.	.	Social quotas	9
Amazon	.	.	Racial quotas	5
Amazon	.	.	Control	14
Amazon	.	Social quotas	.	0
Amazon	.	Racial quotas	.	2
Amazon	.	Control	.	9
Abortion	Amazon	Social quotas	Racial quotas	52
Abortion	Amazon	Racial quotas	Social quotas	61
Abortion	Amazon	Control	Control	102
Abortion	Amazon	.	Social quotas	8
Abortion	Amazon	.	Racial quotas	9
Abortion	Amazon	.	Control	21
Abortion	Amazon	Social quotas	.	5
Abortion	Amazon	Racial quotas	.	4
Abortion	Amazon	Control	.	7
Abortion	Amazon	.	.	29
Abortion	Control	Social quotas	Racial quotas	50
Abortion	Control	Racial quotas	Social quotas	51
Abortion	Control	Control	Control	91
Abortion	Control	.	Social quotas	9
Abortion	Control	.	Racial quotas	8
Abortion	Control	.	Control	16

Abortion	Control	Social quotas	.	4
Abortion	Control	Racial quotas	.	7
Abortion	Control	Control	.	14
Abortion	Control	.	.	27
Abortion	.	.	.	100
Abortion	.	Social quotas	Racial quotas	2
Abortion	.	Racial quotas	Social quotas	3
Abortion	.	Control	Control	27
Abortion	.	.	Social quotas	3
Abortion	.	.	Racial quotas	5
Abortion	.	.	Control	12
Abortion	.	Social quotas	.	3
Abortion	.	Racial quotas	.	2
Abortion	.	Control	.	6
Control	Amazon	Social quotas	Racial quotas	44
Control	Amazon	Racial quotas	Social quotas	51
Control	Amazon	Control	Control	92
Control	Amazon	.	Social quotas	7
Control	Amazon	.	Racial quotas	7
Control	Amazon	.	Control	20
Control	Amazon	Social quotas	.	8
Control	Amazon	Racial quotas	.	7
Control	Amazon	Control	.	12
Control	Amazon	.	.	35
Control	Abortion	Social quotas	Racial quotas	52
Control	Abortion	Racial quotas	Social quotas	52

Control	Abortion	Control	Control	93
Control	Abortion	.	Social quotas	12
Control	Abortion	.	Racial quotas	17
Control	Abortion	.	Control	14
Control	Abortion	Social quotas	.	10
Control	Abortion	Racial quotas	.	1
Control	Abortion	Control	.	5
Control	Abortion	.	.	33
Control	Abortion	Control	Racial quotas	1
Control	Control	Social quotas	Racial quotas	99
Control	Control	Racial quotas	Social quotas	90
Control	Control	Control	Control	191
Control	Control	.	Social quotas	21
Control	Control	.	Racial quotas	15
Control	Control	.	Control	46
Control	Control	Social quotas	.	17
Control	Control	Racial quotas	.	9
Control	Control	Control	.	20
Control	Control	.	.	61
Control	.	Social quotas	Racial quotas	18
Control	.	Racial quotas	Social quotas	5
Control	.	Control	Control	14
Control	.	.	Social quotas	6
Control	.	.	Racial quotas	14
Control	.	.	Control	19
Control	.	Social quotas	.	12

Control	.	Racial quotas	.	5
Control	.	Control	.	5
Control	.	.	.	219
		Total		2931

Note: The table shows all the possible experimental conditions participants could have been assigned to in each wave. In wave 2, participants were randomly assigned to either an abortion or an amazon treatment, which could in turn appear before the DVs (treatment conditions) or after the DVs (control condition). Thus, all participants saw one of the treatments, but those who were presented with the treatment after answering the DVs were in the control condition, whereas those who were presented with the treatment before the DVs were in either one of the treatment conditions (abortion or amazon). We consider participants who answered the DVs first to be in the control condition. In wave 3, those who had been assigned to the abortion (amazon) treatment–regardless of being in the treatment or control condition– were then assigned to the amazon (abortion) treatment, which could again appear before (treatment conditions) or after the DVs (control condition). In wave 4, participants were randomly assigned to a control condition, a social quotas treatment, or a racial quotas treatment. Participants assigned to the control condition in wave 4 was not presented with any text about social or racial quotas. In wave 5, participants who had been assigned to the control condition in wave 4 were then assigned to the same control condition, whereas participants who had been assigned to the social (racial) quotas condition were then assigned to the racial (social) quotas condition. Those who did not participate in wave 4 were randomly assigned to any condition in wave 5. Underlined cases are errors that refer to participants who took paths they should not have taken in waves 4 and 5. These errors occurred because these participants were being dropped from the sample in initial analyses of wave 4 due to incomplete responses, which led them to being randomly assigned to conditions in wave 5. But, following the preregistered exclusion criteria, those participants should not have been dropped, which resulted in these errors.

Supplementary Table 4 | Preregistered results of affective polarization over waves

	Linear Trend for wave												Dummies for each wave											
	Lulistas vs. Bolsonaristas				Left-Right Identification				Anti-Politician Sentiment				Lulistas vs. Bolsonaristas				Left-Right Identification				Anti-Politician Sentiment			
VARIABLES	Liking (1)		Perceived Intellig. (2)		Liking (3)		Perceived Intellig. (4)		Liking (5)		Perceived Intellig. (6)		Liking (7)		Perceived Intellig. (8)		Liking (9)		Perceived Intellig. (10)		Liking (11)		Perceived Intellig. (12)	
	Coeff.	*P-value*	Coeff.	*P-value*	Coeff.	*P-value*	Coeff.	*P-value*	Coeff.	*P-value*	Coeff.	*P-value*	Coeff.	*P-value*	Coeff.	*P-value*	Coeff.	*P-value*	Coeff.	*P-value*	Coeff.	*P-value*	Coeff.	*P-value*
Wave (continuous)	0.84	*0.001*	0.65	*0.006*	0.49	*0.017*	0.17	*0.521*	0.10	*0.783*	-0.07	0.859												
	(0.36, 1.32)		(0.19, 1.12)		(0.88, 0.89)		(-0.35, 0.69)		(-0.59, 0.79)		(-0.80, 0.66)													
Outgroup	-57.85	*< .001*	-53.08	*< .001*	-21.53	*< .001*	-20.9	*< .001*	-38.29	< .001	-35.55	*< .001*	-58.48	*< .001*	-52.84	*< .001*	-23.47	*< .001*	-19.91	*< .001*	-36.65	*< .001*	-33.28	*< .001*
	(-59.93, -55.77)		(-55.09, -51.06)		(-23.19, -19.88)		(-23.00, -18.80)		(-41.24, -35.34)		(-38.58, -32.52)		(-60.67, -56.28)		(-54.99, -50.69)		(-25.34, -21.59)		(-21.35, -18.48)		(-38.73, -34.58)		(-35.33, -31.23)	
Wave X Outgroup	1.76	*< .001*	1.62	*< .001*	0.66	*0.008*	0.36	*0.204*	0.68	*0.080*	0.77	*0.055*												
	(1.12, 2.41)		(0.99, 2.25)		(0.17, 1.15)		(-0.20, 0.91)		(-0.08, 1.43)		(-0.02, 1.56)													
Wave 2													0.97	*0.34*	-0.43	*0.666*	0.83	*0.327*						
													(-1.02, 2.96)		(-2.36, 1.51)		(-0.82, 2.48)							
Wave 3													3.75	*< .001*	2.81	*0.005*	2.57	*0.003*	1.42	*0.088*	0.79	*0.465*	1.47	*0.168*
													(1.69, 5.81)		(0.84, 4.78)		(0.85, 4.30)		(-0.21, 3.05)		(-1.33, 2.91)		(-0.62, 3.55)	
Wave 4													2.99	*0.007*	2.52	*0.018*	0.53	*0.571*	1.13	*0.192*	0.13	*0.909*	0.14	*0.907*
													(0.80, 5.17)		(0.44, 4.61)		(-1.30, 2.36)		(-0.57, 2.83)		(-2.03, 2.29)		(-2.14, 2.41)	
Wave 5													2.04	*0.056*	1.19	*0.248*	1.52	*0.091*	0.54	*0.517*	0.47	*0.662*	0.06	*0.961*
													(-0.06, 4.14)		(-0.83, 3.22)		(-0.24, 3.29)		(-1.09, 2.16)		(-1.65, 2.60)		(-2.15, 2.27)	
Wave 2 X Outgroup													6.68	*< .001*	4.97	*< .001*	5.85	*< .001*						
													(4.06, 9.30)		(2.41, 7.53)		(3.72, 7.98)							
Wave 3 X Outgroup													5.14	*< .001*	3.29	*0.017*	3.06	*0.009*	-0.75	*0.394*	-0.17	*0.887*	-1.24	*0.297*
													(2.32, 7.96)		(0.58, 5.99)		(0.76, 5.36)		(-2.49, 0.98)		(-2.52, 2.18)		(-3.58, 1.09)	
Wave 4 X Outgroup													8.09	*< .001*	6.27	*< .001*	5.14	*< .001*	1.27	*0.168*	1.13	*0.368*	0.70	*0.583*
													(5.08, 11.10)		(3.41, 9.14)		(2.82, 7.45)		(-0.54, 3.07)		(-1.33, 3.60)		(-1.80, 3.21)	
Wave 5 X Outgroup													8.7	*< .001*	7.89	*< .001*	4.43	*< .001*	0.62	*0.487*	1.92	*0.113*	2.16	*0.081*
													(5.82, 11.58)		(5.06, 10.72)		(2.11, 6.75)		(-1.12, 2.36)		(-0.45, 4.29)		(-0.27, 4.59)	
Constant	77.73	*< .001*	74.75	*< .001*	62.13	*< .001*	64.24	*< .001*	71.87	*< .001*	70.35	*< .001*	78.42	< .001	75.61	*< .001*	62.57	*< .001*	64.09	*< .001*	71.87	*< .001*	69.74	*< .001*
	(74.13, 81.32)		(71.18, 78.32)		(58.69, 65.57)		(60.25, 68.23)		(67.62, 76.12)		(66.04, 74.67)		(74.83, 82.02)		(72.05, 79.17)		(59.12, 66.02)		(60.41, 67.77)		(68.10, 75.65)		(65.95, 73.53)	
Control Variables	Yes		Yes		Yes		Yes		Yes		Yes		Yes		Yes		Yes		Yes		Yes		Yes	
Observations	13,594		13,594		11,244		9,332		13,228		13,230		13,594		13,594		11,244		9,332		13,228		13,230	
R-squared	0.52		0.48		0.21		0.21		0.26		0.23		0.52		0.48		0.21		0.21		0.26		0.23	

Notes: For each variable in each model, the table shows coefficients and exact p-values. It also includes 95% confidence intervals in parentheses. Models 1-6 consider continuous time trend, while Models 7-12 consider individual dummies for waves to capture possible non-linearity. Models 1-2 and 7-8 consider lulista versus bolsonarista grouping, whereas models 3-4 and 9-10 consider left-right political self-identification, and models 5-6 and 11-12 anti-politician sentiment. All models were estimated using linear regression models, controlling for participants' age, self-reported sex, household income, education attainment, and region of residence, and dummies for wave-specific treatment conditions. All statistical tests were two-sided.

Supplementary Table 5a | Bayes Factors in the direction of the alternative hypothesis (BF_{10}) and equivalence tests (changes in affective polarization throughout the 2022 election considering the vote intention group definition)

Result	Political group	Dependent variable	BF_{10} and qualitative interpretation	Equivalence test and qualitative interpretation
Changes in affective polarization during the election wave compared to the pre-election period	Vote intention	Liking	Strong evidence (BF_{10}= 0.05) in favour of null	$t(2581) = 3.55$, $p < 0.001$; Effects are statistically equivalent
Changes in affective polarization during the election wave compared to the pre-election period	Vote intention	Intelligence	Strong evidence (BF_{10}= 0.03) in favour of null	$t(2581) = 4.09$, $p < 0.001$; Effects are statistically equivalent
Changes in affective polarization post-election election wave compared to the pre-election period	Vote intention	Liking	Extreme evidence (BF_{10}> 100) in favour of alternative	$t(2581) = -0.06$, $p = 0.524$; No evidence that effects are statistically equivalent
Changes in affective polarization post-election election wave compared to the pre-election period	Vote intention	Intelligence	Very strong evidence (BF_{10}= 53.50) in favour of alternative	$t(2581) = 0.01$, $p = 0.495$; No evidence that effects are statistically equivalent
Changes in ingroup evaluations post-election election wave compared to the pre-election period	Vote intention	Liking	Strong evidence (BF_{10}= 0.04) in favour of null	$t(2581) = -4.02$, $p = 1.000$; No evidence that effects are statistically equivalent
Changes in ingroup evaluations post-election election wave compared to the pre-election period	Vote intention	Intelligence	Strong evidence (BF_{10}= 0.06) in favour of null	$t(2581) = -3.68$, $p = 0.999$; No evidence that effects are statistically equivalent
Changes in outgroup evaluations post-election election wave compared to the pre-election period	Vote intention	Liking	Strong evidence (BF_{10}= 17.07) in favour of alternative	$t(2581) = -4.02$, $p = 1.000$; No evidence that effects are statistically equivalent
Changes in outgroup evaluations post-election election wave compared to the pre-election period	Vote intention	Intelligence	Moderate evidence (BF_{10}= 4.88) in favour of alternative	$t(2581) = -3.68$, $p = 0.999$; No evidence that effects are statistically equivalent

We report Bayes factors in the direction of the alternative for all coefficients of interest. For these analyses, we followed the same approach as in Johnston and Madson (2022)[1] by fitting a Bayesian generalized linear model and calculating the model parameters of interest. For each coefficient, we used the Savage–Dickey density ratio to determine the change in the probability of the null hypothesis from the prior to the posterior distribution by comparing their densities at the null value. Both independent and dependent variables were standardized, and we started with a normal prior distribution for all coefficients with a mean of zero and a standard deviation of 0.50. For all coefficients of interest, we then computed Bayes factors for non-directional hypotheses (two-tailed) using the R package *bayestestR*[2]. We also report qualitative interpretations of the estimated Bayes factors based on Andraszewicz et al. (2015)[3]. For equivalence tests, we conducted one-sided tests (TOST[4]) with the alternative hypothesis that the absolute value of the effect in the population is smaller than the smallest effect size of interest (SESOI). Following pre-registered effect sizes of previous work[5] that similarly examined reductions in affective polarization, we set the SESOI for all equivalence tests at d =.16.

Supplementary Table 5b | Bayes Factors in the direction of the alternative hypothesis (BF_{10}) and equivalence tests (changes in affective polarization throughout the 2022 election considering the ideological left-right self-identification and anti-politician group definitions)

Result	Political group	Dependent variable	BF_{10} and qualitative interpretation	Equivalence test and qualitative interpretation
Changes in affective polarization during the election wave compared to the pre-election period	Left-right self-placement	Liking	Strong evidence (BF_{10}= 0.04) in favour of null	$t(2071) = 3.71$, $p < 0.001$; Effects are statistically equivalent
Changes in affective polarization during the election wave compared to the pre-election period	Left-right self-placement	Intelligence	Strong evidence (BF_{10}= 0.04) in favour of null	$t(1500) = 3.61$, $p < 0.001$; Effects are statistically equivalent
Changes in affective polarization post-election election wave compared to the pre-election period	Left-right self-placement	Liking	Strong evidence (BF_{10}= 0.08) in favour of null	$t(2071) = 1.65$, $p = 0.050$; Effects are statistically equivalent
Changes in affective polarization post-election election wave compared to the pre-election period	Left-right self-placement	Intelligence	Strong evidence (BF_{10}= 0.06) in favour of null	$t(1500) = 1.76$, $p = 0.039$; Effects are statistically equivalent
Changes in affective polarization during the election wave compared to the pre-election period	Anti-politician sentiment	Liking	Strong evidence (BF_{10}= 0.03) in favour of null	$t(2146) = 2.85$, $p = 0.002$; Effects are statistically equivalent
Changes in affective polarization during the election wave compared to the pre-election period	Anti-politician sentiment	Intelligence	Strong evidence (BF_{10}= 0.04) in favour of null	$t(2581) = 2.02$, $p = 0.021$; Effects are statistically equivalent
Changes in affective polarization post-election election wave compared to the pre-election period	Anti-politician sentiment	Liking	Strong evidence (BF_{10}= 0.06) in favour of null	$t(2146) = 3.76$, $p < 0.001$; Effects are statistically equivalent
Changes in affective polarization post-election election wave compared to the pre-election period	Anti-politician sentiment	Intelligence	Strong evidence (BF_{10}= 0.06) in favour of null	$t(2581) = 4.43$, $p < 0.001$; Effects are statistically equivalent

We report Bayes factors in the direction of the alternative for all coefficients of interest. For these analyses, we followed the same approach as in Johnston and Madson (2022)[1] by fitting a Bayesian generalized linear model and calculating the model parameters of interest. For each coefficient, we used the Savage–Dickey density ratio to determine the change in the probability of the null hypothesis from the prior to the posterior distribution by comparing their densities at the null value. Both independent and dependent variables were standardized, and we started with a normal prior distribution for all coefficients with a mean of zero and a standard deviation of 0.50. For all coefficients of interest, we then computed Bayes factors for non-directional hypotheses (two-tailed) using the R package *bayestestR*[2]. We also report qualitative interpretations of the estimated Bayes factors based on Andraszewicz et al. (2015)[3]. For equivalence tests, we conducted one-sided tests (TOST[4]) with the alternative hypothesis that the absolute value of the effect in the population is smaller than the smallest effect size of interest (SESOI). Following pre-registered effect sizes of previous work[5] that similarly examined reductions in affective polarization, we set the SESOI for all equivalence tests at d =.16.

Supplementary Table 5c | Bayes Factors in the direction of the alternative hypothesis (BF_{10}) and equivalence tests (changes in affective meta-polarization throughout the 2022 election considering the vote intention group definition)

Result	Political group	Dependent variable	BF_{10} and qualitative interpretation	Equivalence test and qualitative interpretation
Changes in affective meta-polarization during the election wave compared to the pre-election period	Vote intention	Liking	Strong evidence (BF_{10}= 0.03) in favour of null	$t(3523) = 3.89$, $p < 0.001$; Effects are statistically equivalent
Changes in affective meta-polarization during the election wave compared to the pre-election period	Vote intention	Intelligence	Moderate evidence (BF_{10}= 0.10) in favour of null	$t(2923) = 2.38$, $p = 0.009$; Effects are statistically equivalent
Changes in affective meta-polarization after the elections compared to the pre-election period	Vote intention	Liking	Very strong evidence (BF_{10}= 0.02) in favour of null	$t(3523) = 3.89$, $p < 0.001$; Effects are statistically equivalent
Changes in affective meta-polarization after the elections compared to the pre-election period	Vote intention	Intelligence	Strong evidence (BF_{10}= 0.03) in favour of null	$t(2923) = 3.17$, $p < 0.001$; Effects are statistically equivalent

We report Bayes factors in the direction of the alternative for all coefficients of interest. For these analyses, we followed the same approach as in Johnston and Madson (2022)[1] by fitting a Bayesian generalized linear model and calculating the model parameters of interest. For each coefficient, we used the Savage–Dickey density ratio to determine the change in the probability of the null hypothesis from the prior to the posterior distribution by comparing their densities at the null value. Both independent and dependent variables were standardized, and we started with a normal prior distribution for all coefficients with a mean of zero and a standard deviation of 0.50. For all coefficients of interest, we then computed Bayes factors for non-directional hypotheses (two-tailed) using the R package *bayestestR*[2]. We also report qualitative interpretations of the estimated Bayes factors based on Andraszewicz et al. (2015)[3]. For equivalence tests, we conducted one-sided tests (TOST[4]) with the alternative hypothesis that the absolute value of the effect in the population is smaller than the smallest effect size of interest (SESOI). Following pre-registered effect sizes of previous work[5] that similarly examined reductions in affective polarization, we set the SESOI for all equivalence tests at d =.16.

Supplementary Table 5d | Bayes Factors in the direction of the alternative hypothesis (BF_{10}) and equivalence tests (changes in affective polarization throughout the 2022 FIFA World Cup considering the vote intention group definition)

Result	Political group	Dependent variable	BF_{10} and qualitative interpretation	Equivalence test and qualitative interpretation
Changes in affective polarization after Brazil's victories in the World Cup	Vote intention	Liking	Strong evidence (BF_{10}= 0.05) in favour of null	t(2318) = 2.25, p = 0.012; Effects are statistically equivalent
Changes in affective polarization after Brazil's victories in the World Cup	Vote intention	Intelligence	Strong evidence (BF_{10}= 0.05) in favour of null	t(2316) = 2.17, p = 0.015; Effects are statistically equivalent
Changes in affective polarization after Brazil's defeats in the World Cup	Vote intention	Liking	Strong evidence (BF_{10}= 0.07) in favour of null	t(2318) = 1.65, p = 0.050; Effects are statistically equivalent
Changes in affective polarization after Brazil's defeats in the World Cup	Vote intention	Intelligence	Moderate evidence (BF_{10}= 0.11) in favour of null	t(2316) = 1.32, p = 0.093; No evidence that effects are statistically equivalent
Changes in affective polarization between wave 3 (elections) and the World Cup wave (pre-January 8th)	Vote intention	Liking	Moderate evidence (BF_{10}= 0.18) in favour of null	t(2581) = 1.68, p = 0.046; Effects are statistically equivalent
Changes in affective polarization between wave 3 (elections) and the World Cup wave (pre-January 8th)	Vote intention	Intelligence	Moderate evidence (BF_{10}= 0.25) in favour of null	t(2581) = 1.50, p = 0.067; No evidence that effects are statistically equivalent
Changes in affective polarization between the World Cup wave (pre-January 8th) and the last wave (late January to February)	Vote intention	Liking	Very strong evidence (BF_{10}= 0.02) in favour of null	t(2581) = 3.23, p < 0.001; Effects are statistically equivalent
Changes in affective polarization between the World Cup wave (pre-January 8th) and the last wave (late January to February)	Vote intention	Intelligence	Strong evidence (BF_{10}= 0.04) in favour of null	t(2581) = 2.40, p < 0.001; Effects are statistically equivalent

We report Bayes factors in the direction of the alternative for all coefficients of interest. For these analyses, we followed the same approach as in Johnston and Madson (2022)[1] by fitting a Bayesian generalized linear model and calculating the model parameters of interest. For each coefficient, we used the Savage–Dickey density ratio to determine the change in the probability of the null hypothesis from the prior to the posterior distribution by comparing their densities at the null value. Both independent and dependent variables were standardized, and we started with a normal prior distribution for all coefficients with a mean of zero and a standard deviation of 0.50. For all coefficients of interest, we then computed Bayes factors for non-directional hypotheses (two-tailed) using the R package *bayestestR*[2]. We also report qualitative interpretations of the estimated Bayes factors based on Andraszewicz et al. (2015)[3]. For equivalence tests, we conducted one-sided tests (TOST[4]) with the alternative hypothesis that the absolute value of the effect in the population is smaller than the smallest effect size of interest (SESOI). Following pre-registered effect sizes of previous work[5] that similarly examined reductions in affective polarization, we set the SESOI for all equivalence tests at d =.16.

Supplementary Table 5e | Bayes Factors in the direction of the alternative hypothesis (BF_{10}) and equivalence tests (treatment effects of misperception-correcting information relating to abortion)

Result	Political group	Dependent variable	BF_{10} and qualitative interpretation	Equivalence test and qualitative interpretation
Treatment effects on affective polarization for all participants in waves 2 and 3 (Abortion condition)	Vote intention	Liking	Very strong evidence (BF_{10}= 58.06) in favour of alternative	t(1839) = -0.31, p = 0.622; No evidence that effects are statistically equivalent
Treatment effects on affective polarization for lulistas in waves 2 and 3 (Abortion condition)	Vote intention	Liking	Moderate evidence (BF_{10}= 4.12) in favour of alternative	t(997) = -0.18, p = 0.570; No evidence that effects are statistically equivalent
Treatment effects on affective polarization for bolsonaristas in waves 2 and 3 (Abortion condition)	Vote intention	Liking	Anecdotal evidence (BF_{10}= 2.71) in favour of alternative	t(841) = -0.28, p = 0.610; No evidence that effects are statistically equivalent
Treatment effects on affective polarization for all participants in waves 2 and 3 (Abortion condition)	Vote intention	Intelligence	Extreme evidence (BF_{10}= 114.59) in favour of alternative	t(1839) = -0.84, p = 0.801; No evidence that effects are statistically equivalent
Treatment effects on affective polarization for lulistas in waves 2 and 3 (Abortion condition)	Vote intention	Intelligence	Anecdotal evidence (BF_{10}= 1.61) in favour of alternative	t(997) = 0.01, p = 0.496; No evidence that effects are statistically equivalent
Treatment effects on affective polarization for bolsonaristas in waves 2 and 3 (Abortion condition)	Vote intention	Intelligence	Very strong evidence (BF_{10}= 30.19) in favour of alternative	t(841) = -1.32, p = 0.907; No evidence that effects are statistically equivalent

We report Bayes factors in the direction of the alternative for all coefficients of interest. For these analyses, we followed the same approach as in Johnston and Madson (2022)[1] by fitting a Bayesian generalized linear model and calculating the model parameters of interest. For each coefficient, we used the Savage–Dickey density ratio to determine the change in the probability of the null hypothesis from the prior to the posterior distribution by comparing their densities at the null value. Both independent and dependent variables were standardized, and we started with a normal prior distribution for all coefficients with a mean of zero and a standard deviation of 0.50. For all coefficients of interest, we then computed Bayes factors for non-directional hypotheses (two-tailed) using the R package *bayestestR*[2]. We also report qualitative interpretations of the estimated Bayes factors based on Andraszewicz et al. (2015)[3]. For equivalence tests, we conducted one-sided tests (TOST[4]) with the alternative hypothesis that the absolute value of the effect in the population is smaller than the smallest effect size of interest (SESOI). Following pre-registered effect sizes of previous work[5] that similarly examined reductions in affective polarization, we set the SESOI for all equivalence tests at d =.16.

Supplementary Table 5f | Bayes Factors in the direction of the alternative hypothesis (BF_{10}) and equivalence tests (treatment effects of misperception-correcting information relating to Amazonian deforestation)

Result	Political group	Dependent variable	BF_{10} and qualitative interpretation	Equivalence test and qualitative interpretation
Treatment effects on affective polarization for all participants in waves 2 and 3 (Amazon condition)	Vote intention	Liking	Anecdotal evidence (BF_{10}= 1.35) in favour of alternative	t(1839) = 1.15, p = 0.125; No evidence that effects are statistically equivalent
Treatment effects on affective polarization for lulistas in waves 2 and 3 (Amazon condition)	Vote intention	Liking	Anecdotal evidence (BF_{10}= 0.39) in favour of null	t(997) = 0.65, p = 0.258; No evidence that effects are statistically equivalent
Treatment effects on affective polarization for bolsonaristas in waves 2 and 3 (Amazon condition)	Vote intention	Liking	Moderate evidence (BF_{10}= 0.16) in favour of null	t(841) = 1.04, p = 0.149; No evidence that effects are statistically equivalent
Treatment effects on affective polarization for all participants in waves 2 and 3 (Amazon condition)	Vote intention	Intelligence	Extreme evidence (BF_{10}> 100) in favour of alternative	t(1839) = -0.80, p = 0.787; No evidence that effects are statistically equivalent
Treatment effects on affective polarization for lulistas in waves 2 and 3 (Amazon condition)	Vote intention	Intelligence	Moderate evidence (BF_{10}= 8.48) in favour of alternative	t(997) = -0.74, p = 0.772; No evidence that effects are statistically equivalent
Treatment effects on affective polarization for bolsonaristas in waves 2 and 3 (Amazon condition)	Vote intention	Intelligence	Anecdotal evidence (BF_{10}= 1.84) in favour of alternative	t(841) = -0.25, p = 0.599; No evidence that effects are statistically equivalent

We report Bayes factors in the direction of the alternative for all coefficients of interest. For these analyses, we followed the same approach as in Johnston and Madson (2022)[1] by fitting a Bayesian generalized linear model and calculating the model parameters of interest. For each coefficient, we used the Savage–Dickey density ratio to determine the change in the probability of the null hypothesis from the prior to the posterior distribution by comparing their densities at the null value. Both independent and dependent variables were standardized, and we started with a normal prior distribution for all coefficients with a mean of zero and a standard deviation of 0.50. For all coefficients of interest, we then computed Bayes factors for non-directional hypotheses (two-tailed) using the R package *bayestestR*[2]. We also report qualitative interpretations of the estimated Bayes factors based on Andraszewicz et al. (2015)[3]. For equivalence tests, we conducted one-sided tests (TOST[4]) with the alternative hypothesis that the absolute value of the effect in the population is smaller than the smallest effect size of interest (SESOI). Following pre-registered effect sizes of previous work[5] that similarly examined reductions in affective polarization, we set the SESOI for all equivalence tests at d =.16.

Supplementary Table 5g | Bayes Factors in the direction of the alternative hypothesis (BF_{10}) and equivalence tests (treatment effects of misperception-correcting information relating to social quotas)

Result	Political group	Dependent variable	BF_{10} and qualitative interpretation	Equivalence test and qualitative interpretation
Treatment effects on affective polarization for all participants in waves 4 and 5 (Social quotas condition)	Vote intention	Liking	Strong evidence (BF_{10}= 0.05) in favour of null	t(1437) = 2.67, p = 0.004; Effects are statistically equivalent
Treatment effects on affective polarization for lulistas in waves 4 and 5 (Social quotas condition)	Vote intention	Liking	Strong evidence (BF_{10}= 0.07) in favour of null	t(776) = 1.77, p = 0.038; Effects are statistically equivalent
Treatment effects on affective polarization for bolsonaristas in waves 4 and 5 (Social quotas condition)	Vote intention	Liking	Strong evidence (BF_{10}= 0.05) in favour of null	t(660) = 2.02, p = 0.022; Effects are statistically equivalent
Treatment effects on affective polarization for all participants in waves 4 and 5 (Social quotas condition)	Vote intention	Intelligence	Moderate evidence (BF_{10}= 0.14) in favour of null	t(1437) = 2.01, p = 0.023; Effects are statistically equivalent
Treatment effects on affective polarization for lulistas in waves 4 and 5 (Social quotas condition)	Vote intention	Intelligence	Moderate evidence (BF_{10}= 0.31) in favour of null	t(776) = 0.76, p = 0.224; No evidence that effects are statistically equivalent
Treatment effects on affective polarization for bolsonaristas in waves 4 and 5 (Social quotas condition)	Vote intention	Intelligence	Strong evidence (BF_{10}= 0.05) in favour of null	t(660) = 2.20, p = 0.014; Effects are statistically equivalent

We report Bayes factors in the direction of the alternative for all coefficients of interest. For these analyses, we followed the same approach as in Johnston and Madson (2022)[1] by fitting a Bayesian generalized linear model and calculating the model parameters of interest. For each coefficient, we used the Savage–Dickey density ratio to determine the change in the probability of the null hypothesis from the prior to the posterior distribution by comparing their densities at the null value. Both independent and dependent variables were standardized, and we started with a normal prior distribution for all coefficients with a mean of zero and a standard deviation of 0.50. For all coefficients of interest, we then computed Bayes factors for non-directional hypotheses (two-tailed) using the R package *bayestestR*[2]. We also report qualitative interpretations of the estimated Bayes factors based on Andraszewicz et al. (2015)[3]. For equivalence tests, we conducted one-sided tests (TOST[4]) with the alternative hypothesis that the absolute value of the effect in the population is smaller than the smallest effect size of interest (SESOI). Following pre-registered effect sizes of previous work[5] that similarly examined reductions in affective polarization, we set the SESOI for all equivalence tests at d =.16.

Supplementary Table 5h | Bayes Factors in the direction of the alternative hypothesis (BF_{10}) and equivalence tests (treatment effects of misperception-correcting information relating to racial quotas)

Result	Political group	Dependent variable	BF_{10} and qualitative interpretation	Equivalence test and qualitative interpretation
Treatment effects on affective polarization for all participants in waves 4 and 5 (Racial quotas condition)	Vote intention	Liking	Moderate evidence (BF_{10}= 0.17) in favour of null	t(1437) = 2.17, p = 0.015; Effects are statistically equivalent
Treatment effects on affective polarization for lulistas in waves 4 and 5 (Racial quotas condition)	Vote intention	Liking	Strong evidence (BF_{10}= 0.07) in favour of null	t(776) = 1.83, p = 0.034; Effects are statistically equivalent
Treatment effects on affective polarization for bolsonaristas in waves 4 and 5 (Racial quotas condition)	Vote intention	Liking	Moderate evidence (BF_{10}= 0.13) in favour of null	t(660) = 1.20, p = 0.115; No evidence that effects are statistically equivalent
Treatment effects on affective polarization for all participants in waves 4 and 5 (Racial quotas condition)	Vote intention	Intelligence	Anecdotal evidence (BF_{10}= 0.80) in favour of null	t(1437) = 1.08, p = 0.141; No evidence that effects are statistically equivalent
Treatment effects on affective polarization for lulistas in waves 4 and 5 (Racial quotas condition)	Vote intention	Intelligence	Anecdotal evidence (BF_{10}= 1.01) in favour of alternative	t(776) = 0.30, p = 0.381; No evidence that effects are statistically equivalent
Treatment effects on affective polarization for bolsonaristas in waves 4 and 5 (Racial quotas condition)	Vote intention	Intelligence	Strong evidence (BF_{10}= 0.10) in favour of null	t(660) = 1.32, p = 0.094; No evidence that effects are statistically equivalent

We report Bayes factors in the direction of the alternative for all coefficients of interest. For these analyses, we followed the same approach as in Johnston and Madson (2022)[1] by fitting a Bayesian generalized linear model and calculating the model parameters of interest. For each coefficient, we used the Savage–Dickey density ratio to determine the change in the probability of the null hypothesis from the prior to the posterior distribution by comparing their densities at the null value. Both independent and dependent variables were standardized, and we started with a normal prior distribution for all coefficients with a mean of zero and a standard deviation of 0.50. For all coefficients of interest, we then computed Bayes factors for non-directional hypotheses (two-tailed) using the R package *bayestestR*[2]. We also report qualitative interpretations of the estimated Bayes factors based on Andraszewicz et al. (2015)[3]. For equivalence tests, we conducted one-sided tests (TOST[4]) with the alternative hypothesis that the absolute value of the effect in the population is smaller than the smallest effect size of interest (SESOI). Following pre-registered effect sizes of previous work[5] that similarly examined reductions in affective polarization, we set the SESOI for all equivalence tests at d =.16.

Supplementary Table 5i | Bayes Factors in the direction of the alternative hypothesis (BF_{10}) and equivalence tests (treatment effects of misperception-correcting information on liking scores across waves)

Result	Political group	Dependent variable	BF_{10} and qualitative interpretation	Equivalence test and qualitative interpretation
Treatment effects on affective polarization across waves 2 and 3 (Abortion condition)	Vote intention	Liking	Strong evidence (BF_{10}= 0.06) in favour of null	$t(1839) = 4.03$, $p < 0.001$; Effects are statistically equivalent
Treatment effects on affective polarization across waves 2 and 3 (Amazon condition)	Vote intention	Liking	Strong evidence (BF_{10}= 0.04) in favour of null	$t(1839) = 4.33$, $p < 0.001$; Effects are statistically equivalent
Treatment effects on affective polarization across waves 4 and 5 (Social quotas condition)	Vote intention	Liking	Strong evidence (BF_{10}= 0.05) in favour of null	$t(1437) = 4.83$, $p < 0.001$; Effects are statistically equivalent
Treatment effects on affective polarization across waves 4 and 5 (Racial quotas condition)	Vote intention	Liking	Moderate evidence (BF_{10}= 3.17) in favour of alternative	$t(1437) = 7.72$, $p < 0.001$; Effects are statistically equivalent
Treatment effects on affective polarization for all participants in wave 4 (Racial quotas condition)	Vote intention	Liking	Anecdotal evidence (BF_{10}= 2.96) in favour of alternative	$t(1437) = -0.50$, $p = 0.692$; No evidence that effects are statistically equivalent
Treatment effects on affective polarization for all participants in wave 5 (Racial quotas condition)	Vote intention	Liking	Strong evidence (BF_{10}= 0.07) in favour of null	$t(1275) = 3.61$, $p < 0.001$; Effects are statistically equivalent

We report Bayes factors in the direction of the alternative for all coefficients of interest. For these analyses, we followed the same approach as in Johnston and Madson (2022)[1] by fitting a Bayesian generalized linear model and calculating the model parameters of interest. For each coefficient, we used the Savage–Dickey density ratio to determine the change in the probability of the null hypothesis from the prior to the posterior distribution by comparing their densities at the null value. Both independent and dependent variables were standardized, and we started with a normal prior distribution for all coefficients with a mean of zero and a standard deviation of 0.50. For all coefficients of interest, we then computed Bayes factors for non-directional hypotheses (two-tailed) using the R package *bayestestR*[2]. We also report qualitative interpretations of the estimated Bayes factors based on Andraszewicz et al. (2015)[3]. For equivalence tests, we conducted one-sided tests (TOST[4]) with the alternative hypothesis that the absolute value of the effect in the population is smaller than the smallest effect size of interest (SESOI). Following pre-registered effect sizes of previous work[5] that similarly examined reductions in affective polarization, we set the SESOI for all equivalence tests at d =.16.

Supplementary Table 5j | Bayes Factors in the direction of the alternative hypothesis (BF_{10}) and equivalence tests (treatment effects of misperception-correcting information on perceived intelligence scores across waves)

Result	Political group	Dependent variable	BF_{10} and qualitative interpretation	Equivalence test and qualitative interpretation
Treatment effects on affective polarization across waves 2 and 3 (Abortion condition)	Vote intention	Intelligence	Strong evidence (BF_{10}= 0.07) in favour of null	t(1839) = 3.63, p < 0.001; Effects are statistically equivalent
Treatment effects on affective polarization across waves 2 and 3 (Amazon condition)	Vote intention	Intelligence	Strong evidence (BF_{10}= 0.08) in favour of null	t(1839) = 3.57, p < 0.001; Effects are statistically equivalent
Treatment effects on affective polarization across waves 4 and 5 (Social quotas condition)	Vote intention	Intelligence	Strong evidence (BF_{10}= 0.05) in favour of null	t(1437) = 4.42, p < 0.001; Effects are statistically equivalent
Treatment effects on affective polarization across waves 4 and 5 (Racial quotas condition)	Vote intention	Intelligence	Anecdotal evidence (BF_{10}= 0.63) in favour of null	t(1437) = 6.92, p < 0.001; Effects are statistically equivalent
Treatment effects on affective polarization for all participants in wave 4 (Racial quotas condition)	Vote intention	Intelligence	Moderate evidence (BF_{10}= 4.62) in favour of alternative	t(1437) = -0.88, p = 0.811; No evidence that effects are statistically equivalent
Treatment effects on affective polarization for all participants in wave 5 (Racial quotas condition)	Vote intention	Intelligence	Strong evidence (BF_{10}= 0.05) in favour of null	t(1275) = 2.37, p < 0.001; Effects are statistically equivalent

We report Bayes factors in the direction of the alternative for all coefficients of interest. For these analyses, we followed the same approach as in Johnston and Madson (2022)[1] by fitting a Bayesian generalized linear model and calculating the model parameters of interest. For each coefficient, we used the Savage–Dickey density ratio to determine the change in the probability of the null hypothesis from the prior to the posterior distribution by comparing their densities at the null value. Both independent and dependent variables were standardized, and we started with a normal prior distribution for all coefficients with a mean of zero and a standard deviation of 0.50. For all coefficients of interest, we then computed Bayes factors for non-directional hypotheses (two-tailed) using the R package *bayestestR*[2]. We also report qualitative interpretations of the estimated Bayes factors based on Andraszewicz et al. (2015)[3]. For equivalence tests, we conducted one-sided tests (TOST[4]) with the alternative hypothesis that the absolute value of the effect in the population is smaller than the smallest effect size of interest (SESOI). Following pre-registered effect sizes of previous work[5] that similarly examined reductions in affective polarization, we set the SESOI for all equivalence tests at d =.16.

Supplementary Table 5k | Bayes Factors in the direction of the alternative hypothesis (BF_{10}) and equivalence tests (treatment effects of misperception-correcting information on policy support for abortion and Amazonian deforestation)

Result	Political group	Dependent variable	BF_{10} and qualitative interpretation	Equivalence test and qualitative interpretation
Treatment effects on issue polarization (Abortion condition)	Vote intention	Policy support	Strong evidence (BF_{10}= 20.11) in favour of alternative	$t(1798) = 0.33$, $p = 0.327$; No evidence that effects are statistically equivalent
Treatment effects on policy support for lulistas (Abortion condition)	Vote intention	Policy support	Extreme evidence (BF_{10}= 172.41) in favour of alternative	$t(1798) = -0.24$, $p = 0.594$; No evidence that effects are statistically equivalent
Treatment effects on policy support for bolsonaristas (Abortion condition)	Vote intention	Policy support	Strong evidence (BF_{10}= 0.03) in favour of null	$t(1798) = 1.15$, $p = 0.125$; No evidence that effects are statistically equivalent
Treatment effects on issue polarization (Amazon condition)	Vote intention	Policy support	Anecdotal evidence (BF10= 1.15) in favour of alternative	$t(1798) = 0.03$, $p = 0.487$; No evidence that effects are statistically equivalent
Treatment effects on policy support for lulistas (Amazon condition)	Vote intention	Policy support	Anecdotal evidence (BF_{10}= 1.17) in favour of alternative	$t(1798) = 0.12$, $p = 0.452$; No evidence that effects are statistically equivalent
Treatment effects on policy support for bolsonaristas (Amazon condition)	Vote intention	Policy support	Strong evidence (BF_{10}= 0.04) in favour of null	$t(1798) = 1.48$, $p = 0.069$; No evidence that effects are statistically equivalent
Treatment effects on issue polarization across waves 2 and 3 (Abortion condition)	Vote intention	Policy support	Strong evidence (BF_{10}= 0.04) in favour of null	$t(1798) = 2.12$, $p = 0.017$; Effects are statistically equivalent
Treatment effects on issue polarization across waves 2 and 3 (Amazon condition)	Vote intention	Policy support	Strong evidence (BF_{10}= 0.06) in favour of null	$t(1798) = 1.88$, $p = 0.030$; Effects are statistically equivalent

We report Bayes factors in the direction of the alternative for all coefficients of interest. For these analyses, we followed the same approach as in Johnston and Madson (2022)[1] by fitting a Bayesian generalized linear model and calculating the model parameters of interest. For each coefficient, we used the Savage–Dickey density ratio to determine the change in the probability of the null hypothesis from the prior to the posterior distribution by comparing their densities at the null value. Both independent and dependent variables were standardized, and we started with a normal prior distribution for all coefficients with a mean of zero and a standard deviation of 0.50. For all coefficients of interest, we then computed Bayes factors for non-directional hypotheses (two-tailed) using the R package *bayestestR*[2]. We also report qualitative interpretations of the estimated Bayes factors based on Andraszewicz et al. (2015)[3]. For equivalence tests, we conducted one-sided tests (TOST[4]) with the alternative hypothesis that the absolute value of the effect in the population is smaller than the smallest effect size of interest (SESOI). Following pre-registered effect sizes of previous work[5] that similarly examined reductions in affective polarization, we set the SESOI for all equivalence tests at d =.16.

Supplementary Table 5l | Bayes Factors in the direction of the alternative hypothesis (BF_{10}) and equivalence tests (treatment effects of misperception-correcting information on policy support for social and racial quotas)

Result	Political group	Dependent variable	BF_{10} and qualitative interpretation	Equivalence test and qualitative interpretation
Treatment effects on issue polarization (Social quotas condition)	Vote intention	Policy support	Strong evidence (BF_{10}= 0.04) in favour of null	t(1424) = 1.41, p = 0.080; No evidence that effects are statistically equivalent
Treatment effects on policy support for lulistas (Social quotas condition)	Vote intention	Policy support	Moderate evidence (BF_{10}= 0.14) in favour of null	t(1424) = 0.37, p = 0.355; No evidence that effects are statistically equivalent
Treatment effects on policy support for bolsonaristas (Social quotas condition)	Vote intention	Policy support	Strong evidence (BF_{10}= 0.07) in favour of null	t(1424) = 0.32, p = 0.376; No evidence that effects are statistically equivalent
Treatment effects on issue polarization (Racial quotas condition)	Vote intention	Policy support	Strong evidence (BF_{10}= 0.03) in favour of null	t(1424) = 1.28, p = 0.100; No evidence that effects are statistically equivalent
Treatment effects on policy support for lulistas (Racial quotas condition)	Vote intention	Policy support	Anecdotal evidence (BF10= 0.92) in favour of null	t(1424) = -0.82, p = 0.793; No evidence that effects are statistically equivalent
Treatment effects on policy support for bolsonaristas (Racial quotas condition)	Vote intention	Policy support	Strong evidence (BF_{10}= 2.27) in favour of alternative	t(1424) = -0.61, p = 0.728; No evidence that effects are statistically equivalent
Treatment effects on issue polarization across waves 4 and 5 (Social quotas condition)	Vote intention	Policy support	Anecdotal evidence (BF_{10}= 0.75) in favour of null	t(1424) = -0.20, p = 0.580; No evidence that effects are statistically equivalent
Treatment effects on issue polarization across waves 4 and 5 (Racial quotas condition)	Vote intention	Policy support	Strong evidence (BF_{10}= 0.04) in favour of null	t(1424) = 1.42, p = 0.078; No evidence that effects are statistically equivalent

We report Bayes factors in the direction of the alternative for all coefficients of interest. For these analyses, we followed the same approach as in Johnston and Madson (2022)[1] by fitting a Bayesian generalized linear model and calculating the model parameters of interest. For each coefficient, we used the Savage–Dickey density ratio to determine the change in the probability of the null hypothesis from the prior to the posterior distribution by comparing their densities at the null value. Both independent and dependent variables were standardized, and we started with a normal prior distribution for all coefficients with a mean of zero and a standard deviation of 0.50. For all coefficients of interest, we then computed Bayes factors for non-directional hypotheses (two-tailed) using the R package *bayestestR*[2]. We also report qualitative interpretations of the estimated Bayes factors based on Andraszewicz et al. (2015)[3]. For equivalence tests, we conducted one-sided tests (TOST[4]) with the alternative hypothesis that the absolute value of the effect in the population is smaller than the smallest effect size of interest (SESOI). Following pre-registered effect sizes of previous work[5] that similarly examined reductions in affective polarization, we set the SESOI for all equivalence tests at d =.16.

Supplementary Table 5m | Bayes Factors in the direction of the alternative hypothesis (BF_{10}) and equivalence tests (changes in political identity salience throughout the 2022 election)

Result	Political group	Dependent variable	BF_{10} and qualitative interpretation	Equivalence test and qualitative interpretation
Reduction in political identity salience post-election election wave compared to the election period	Pro-politician group	Identity salience	Moderate evidence (BF_{10}= 5.24) in favour of alternative	t(4242) = -3.16, p = 0.999; No evidence that effects are statistically equivalent

We report Bayes factors in the direction of the alternative for all coefficients of interest. For these analyses, we followed the same approach as in Johnston and Madson (2022)[1] by fitting a Bayesian generalized linear model and calculating the model parameters of interest. For each coefficient, we used the Savage–Dickey density ratio to determine the change in the probability of the null hypothesis from the prior to the posterior distribution by comparing their densities at the null value. Both independent and dependent variables were standardized, and we started with a normal prior distribution for all coefficients with a mean of zero and a standard deviation of 0.50. For all coefficients of interest, we then computed Bayes factors for non-directional hypotheses (two-tailed) using the R package *bayestestR*[2]. We also report qualitative interpretations of the estimated Bayes factors based on Andraszewicz et al. (2015)[3]. For equivalence tests, we conducted one-sided tests (TOST[4]) with the alternative hypothesis that the absolute value of the effect in the population is smaller than the smallest effect size of interest (SESOI). Following pre-registered effect sizes of previous work[5] that similarly examined reductions in affective polarization, we set the SESOI for all equivalence tests at d =.16.

Supplementary Table 6 | Actual and perceived policy support among bolsonaristas and lulistas

Policy	Respondent	Actual Support	Perceived Support	
			Lulistas	Bolsonaristas
Amazon	Lulista	79.91% [76.85%, 82.97%]	69.41% [67.38%, 71.44%]	34.60% [32.19%, 37.02%]
	Bolsonarista	63.73% [59.91%, 67.54%]	49.07% [46.57%, 51.59%]	63.40% [61.13%, 65.67%]
Abortion	Lulista	46.37% [42.57%, 50.18%]	59.78% [57.99%, 61.57%]	26.28% [24.06%, 28.49%]
	Bolsonarista	10.95% [8.47%, 13.43%]	81.48% [79.64%, 83.32%]	23.96% [21.80%, 26.12%]
Social Quotas	Lulista	93.42% [92.29%, 94.56%]	83.66% [81.58%, 85.75%]	27.09% [24.16%, 30.02%]
	Bolsonarista	79.38% [77.45%, 81.32%]	71.29% [67.70%, 74.87%]	67.68% [64.56%, 70.80%]
Racial Quotas	Lulista	88.33% [86.85%, 89.80%]	83.78% [81.72%, 85.84%]	23.53% [20.61%, 26.45%]
	Bolsonarista	48.19% [45.80%, 50.58%]	77.91% [74.80%, 81.02%]	50.77% [47.16%, 54.38%]

Supplementary Table 7 | Effects of misperception-correcting information on liking and perceived intelligence scores

	Waves 2 and 3								Waves 4 and 5							
VARIABLES	Liking (1)		Liking (2)		Perceived Intellig. (3)		Perceived Intellig. (4)		Liking (5)		Liking (6)		Perceived Intellig. (7)		Perceived Intellig. (8)	
	Coeff.	*P-value*	Coeff.	*P-value*	Coeff.	*P-value*	Coeff.	`	Coeff.	*P-value*	Coeff.	*P-value*	Coeff.	*P-value*	Coeff.	*P-value*
Abortion	-		-		-		-									
Amazon	-		-		-		-									
Social Quotas									-		-		-		-	
Racial Quotas									-		-		-		-	
Ingroup	55.30	*< .001*	52.78	*< .001*	52.27	*< .001*	50.2	*< .001*	51.4	*< .001*	49.28	*< .001*	47.6	*< .001*	46.4	*< .001*
	(53.14, 57.46)		(50.49, 55.07)		(50.17, 54.37)		(48.02, 52.37)		(48.64, 54.16)		(46.67, 51.89)		(44.83, 50.38)		(43.84, 48. 96)	
Abortion X Ingroup	-6.66	*< .001*	-6.20	*0.002*	-7.18	*< .001*	-6.05	*0.001*								
	(-9.97, -3.35)		(-10.11, -2.29)		(-10,32, -4.04)		(-9.79, -2.32)									
Amazon X Ingroup	-4.32	*0.009*	-3.99	*0.042*	-7.08	*< .001*	-6.04	*0.002*								
	(-7.54, -1.09)		(-7.85, -0.14)		(-10.22, -3.95)		(-9.78, -2.31)									
Social Quotas X Ingroup									-2.13	*0.324*	-2.98	*0.197*	-3.00	*0.156*	-3.86	*0.082*
									(-6.36, 2.10)		(-7.51, 1.55)		(-7.16, 1.15)		(-8.20, 0.49)	
Racial Quotas X Ingroup									-3.07	*0.151*	-3.74	*0.099*	-4.71	*0.028*	-5.7	*0.012*
									(-7.26, 1.13)		(-8.18, 0.70)		(-8.91, -0.50)		(-10.13, -1.26)	
Constant	21.19	< .001	23.03	*< .001*	23.09	*< .001*	24.47	*< .001*	24.03	*< .001*	26.1	*< .001*	26.17	*< .001*	27.98	*< .001*
	(20.37, 22.02)		(22.24, 23.82)		(22.28, 23.89)		(23.72, 25.23)		(23.05, 25.01)		(25.18, 27.01)		(25.19, 27.14)		(27.08, 28.87)	
Fixed Effects	Yes		Yes		Yes		Yes		Yes		Yes		Yes		Yes	
Control Variables	No		Yes		No		Yes		No		Yes		No		Yes	
Observations	6,530		4,942		6,530		4,942		4,912		3,716		4,912		3,716	
R-squared	0.63		0.63		0.61		0.63		0.61		0.61		0.57		0.58	
Number of id	3,265		2,471		3,265		2,471		2,456		1,858		2,456		1,858	

Notes: For each variable in each model, the table shows coefficients and exact p-values. It also includes 95% confidence intervals in parentheses. All models are calculated considering the lulista versus bolsonarista grouping. Models 1-4 examine effects of the Abortion- and Amazon-related treatments fielded in waves 2 and 3 on liking and perceived intelligence, while Models 5-8 examine effects of the Social quotas- and Racial quotas-related treatments fielded in waves 4 and 5 on the same outcomes. All models were estimated using linear regression models and include fixed effects. Simple main effects of treatment conditions are therefore not available because the fixed effects capture all the between-subject variability. Only interactions between treatment and the ingroup dummy are included. Control variables include covariates for the interactions between outgroup and age (standardized), gender (effects-coded), interest in politics (standardized), religiosity (standardized) and educational level (standardized). All statistical tests are two-sided.

Supplementary Table 8 | Effects of misperception-correcting information on policy support

VARIABLES	Support for Abortion		Support for Zero Amazon Deforestation		Support for Social Quotas		Support for Racial Quotas	
	Coeff.	*P-value*	Coeff.	*P-value*	Coeff.	*P-value*	Coeff.	*P-value*
Lulista (vs. Bolsonarista)	2.20	*< .001*	0.90	*< .001*	1.22	*< .001*	2.49	*< .001*
	(2.03, 2.37)		(0.74, 1.05)		(1.05, 1.38)		(2.31, 2.67)	
Post	0.03	*0.564*	0.06	*0.377*	0.08	*0.425*	0.21	*0.031*
	(-0.06, 0.12)		(-0.07, 0.18)		(-0.11, 0.27)		(0.02, 0.39)	
Lulista X Post	-0.24	*< .001*	-0.22	*0.009*	-0.01	*0.936*	-0.05	*0.662*
	(-0.36, -0.12)		(-0.39, -0.05)		(-0.25, 0.23)		(-0.29, 0.18)	
Constant	1.12	*< .001*	4.64	*< .001*	5.14	*< .001*	3.47	*< .001*
	(0.60, 1.64)		(4.18, 5.10)		(4.62, 5.66)		(2.88, 4.05)	
Control Variables	Yes		Yes		Yes		Yes	
Observations	4,733		4,744		3,030		3,011	
R-squared	0.29		0.06		0.15		0.38	

Notes: For each variable in each model, the table shows coefficients and exact p-values. It also includes 95% confidence intervals in parentheses. All models are calculated considering the lulista versus bolsonarista grouping. All models were estimated using linear regression models and include control variables for age, sex, education, and income. All statistical tests are two-sided.

Supplementary Table 9 | Benjamin-Hochberg correction for multiple hypotheses testing

			Vote intention		Left-Right		Anti-politician sentiment	
			P-value	Adjusted P-value	P-value	Adjusted P-value	P-value	Adjusted P-value
Over time	During election	Liking	0.266	0.399	0.559	0.671	0.877	0.877
	Post-election		0.001	0.006	0.086	0.258	0.147	0.294
	During election	Intelligence	0.728	0.728	0.394	0.473	0.297	0.446
	Post-election		0.001	0.006	0.232	0.446	0.181	0.446
Abortion	Liking		0.001	0.002	0.001	0.002	0.003	0.003
	Intelligence		0.001	0.001	0.001	0.001	0.001	0.001
	Policy Support		0.001	0.001	0.001	0.001	0.001	0.001
Amazon	Liking		0.009	0.014	0.007	0.014	0.020	0.020
	Intelligence		0.001	0.014	0.001	0.014	0.007	0.007
	Policy Support		0.012	0.018	0.023	0.023	0.007	0.018
Social Quotas	Liking		0.324	0.363	0.363	0.363	0.151	0.363
	Intelligence		0.156	0.266	0.494	0.494	0.177	0.266
	Policy Support		0.977	0.991	0.991	0.991	0.638	0.991
Racial Quotas	Liking		0.151	0.226	0.617	0.617	0.048	0.144
	Intelligence		0.028	0.084	0.861	0.861	0.111	0.167
	Policy Support		0.654	0.654	0.299	0.606	0.404	0.606

Notes: For each key hypothesis, the table shows the reported p-value that is used as criteria for statistical significance, as well as the adjusted p-value from the Benjamin-Hochberg correction. All statistical tests are two-sided.

Supplementary Figures

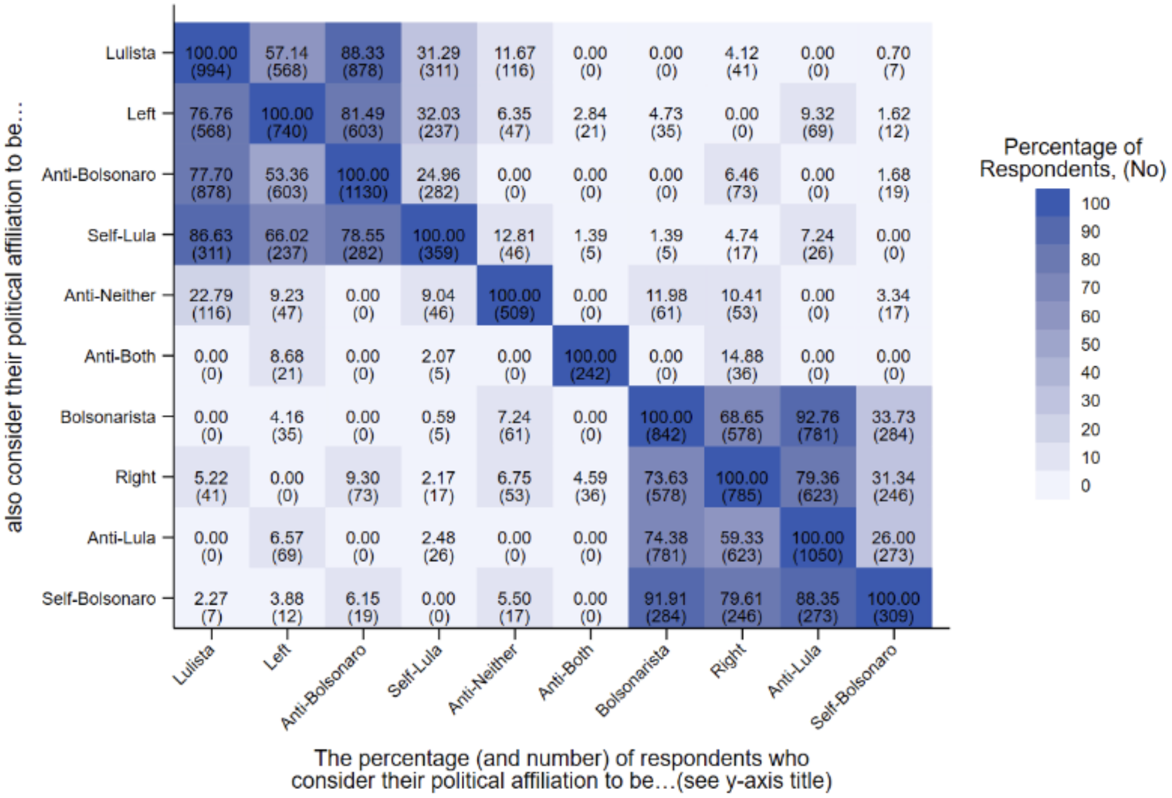

Supplementary Fig. 1 | Co-occurrence of political affiliation considering alternative grouping criteria.

The figure shows the number and percentage of respondents who identify themselves as part of different groups concomitantly. For example, 568 respondents identify as both lulistas and left-wing. In percentage terms, this corresponds to 76% of lulistas identifying as left-wing. lulista and bolsonarista are defined according to intention to vote for the respective candidate in the first round of the presidential election as reported in wave 2. Left and Right are defined according to self-reported identification on the political spectrum as reported in wave 2. Anti-Bolsonaro, anti-Lula, anti-neither, and anti-both are defined according to participants' indication that they would never vote for Bolsonaro, Lula, neither of these candidates, or either of these candidates, respectively, as reported in wave 2. And Self-Lula and Self-Bolsonaro refer to self-reported identification as a lulista or a bolsonarista.

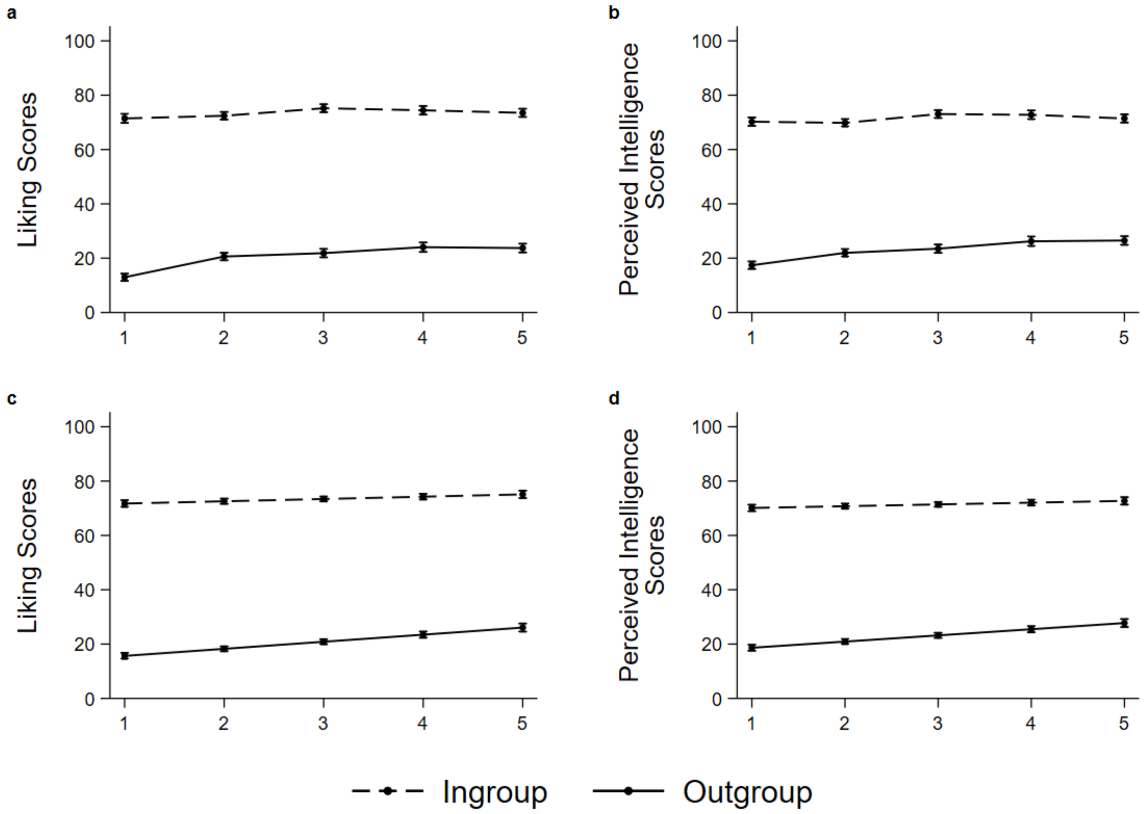

Supplementary Fig. 2 | Evolution of affective polarization over waves considering preregistered models, using first-round election intention-to-vote definition of pro-person groups (lulistas and bolsonaristas).

Panel a (N = 13,594) depicts how average liking scores fluctuate over the five-wave period for political ingroups (individuals who share the respondent's political self-categorization) and outgroups (individuals who hold a different political self-categorization). Panel b (N = 13,594) depicts equivalent effects for perceived intelligence. Panels c (N = 13,594) and d (N = 13,594) display the respective liking and perceived intelligence scores considering linear time trends instead of dummies for individual waves. As preregistered, these effects were estimated using linear regression models, controlling for participants' age, gender, household income, educational attainment, region of residence, and dummies for wave-specific treatment conditions. To mitigate endogeneity concerns (i.e., participants changing both their political identification and affective polarization scores over time due to unobserved factors), we used as the grouping criterion the participants' voting intentions reported in wave 2. Models exclude participants who did not report intentions to vote for either Lula or Bolsonaro and were estimated with clustered standard errors at the individual level. Data are presented as model-predicted mean values, with error bars indicating 95% confidence intervals. All statistical tests were two-sided.

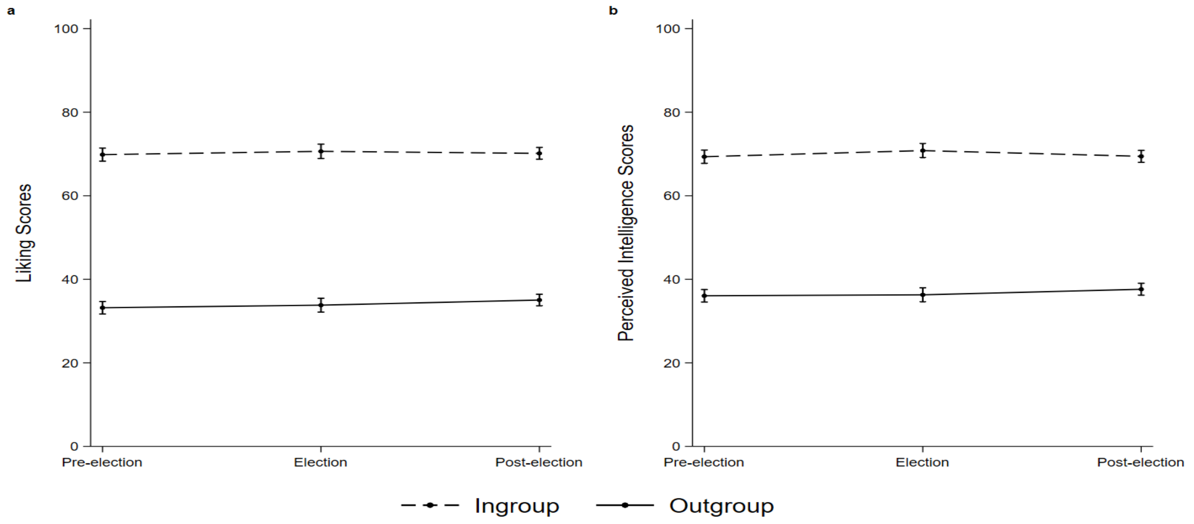

Supplementary Fig. 3 | Evolution of affective polarization using anti-Lula and anti-Bolsonaro sentiment.

Panels a (N = 13,228) depicts how average liking scores fluctuate before, during, and after the election period for political ingroups (individuals who share the respondent's political self-categorization) and outgroups (individuals who hold a different political self-categorization). Panel b (N = 13,230) depicts analogous effects for perceived intelligence. These effects were estimated using linear regression models, controlling for participants' age, gender, household income, educational attainment, region of residence, and dummies for wave-specific treatment conditions. This figure considers as the grouping criterion those who stated they would never vote for Lula/Bolsonaro in the first-round of the elections. To mitigate endogeneity concerns (i.e., participants changing both their political identification and affective polarization scores over time due to unobserved factors), we used as grouping criterion the participants' anti-politician sentiment reported in wave 2. The panels exclude participants who either did not express anti-sentiment towards either Lula or Bolsonaro or who reported anti-sentiment towards both figures, and were estimated with clustered standard errors at the individual level. Data are presented as model-predicted mean values, with error bars indicating 95% confidence intervals. All statistical tests were two-sided. There was no evidence of changes in affective polarization during the election compared to the pre-election period, both in terms of liking (b = 0.17, $t(2,146) = 0.14$, $p = 0.887$, 95% CI [-2.18, -2.52]) and perceived intelligence (b = 1.24, $t(2,146) = 1.04$, $p = 0.297$, 95% CI [-1.09, -3.58]). The TOST procedure indicated that these effects were statistically equivalent to the SESOI (liking: $t(2,146) = 2.85$, $p = 0.002$; perceived intelligence: $t(2,146) = 2.02$, $p = 0.021$). Results for the post-election (vs. pre-election) period were non-significant for liking (b = -1.54, $t(2,146) = -1.45$, $p = 0.147$, 95% CI [-3.62, 0.54], standardized coefficient = -0.01) and perceived intelligence (b = -1.46, $t(2,146) = -1.34$, $p = 0.181$, 95% CI [-3.60, 0.68], standardized coefficient = 0.01). But while equivalence tests demonstrate statistical equivalence for liking post-election ($t(2,146) = 1.87$, $p = 0.031$), there is no evidence that the change is smaller than the SESOI in the population ($t(2,146) = .1.06$, $p = 0.146$).

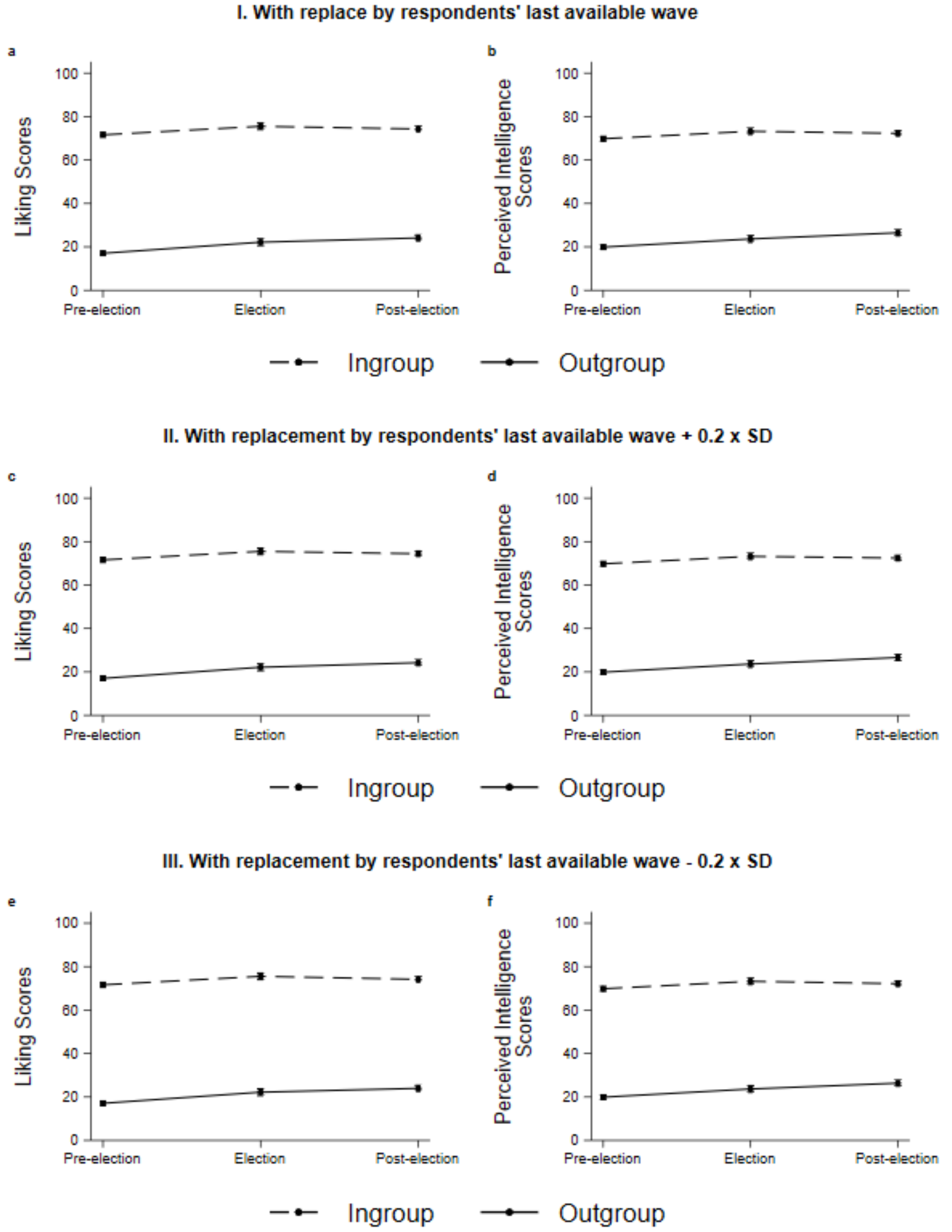

Supplementary Fig. 4 | Sensitivity analyses showing evolution of affective polarization for lulistas and bolsonaristas with replacement of missing values by values along the interval [-.20 SD, + .20 SD] of the respondents' liking and perceived intelligence scores reported in the last wave they had provided such scores.

Panel a (N = 13,702) depicts how average liking scores fluctuate before, during, and after the election period for political ingroups (individuals who share the respondent's political self-categorization) and outgroups (individuals who hold a different political self-categorization),

replacing missing linking score values by the score reported in the last wave they had provided. Panel b (N = 13,702) depicts equivalent effects for perceived intelligence. Panel c (N = 13,702) depicts how average liking scores fluctuate before, during, and after the election period for political ingroups and outgroups, replacing missing linking score values by the score reported in the last they had provided, adjusting it by an increment equivalent to 0.2 standard deviations derived from the variability in changing liking scores. Panel d (N = 13,702) depicts equivalent effects for perceived intelligence. Panel e (N = 13,702) depicts how average liking scores fluctuate before, during, and after the election period for political ingroups and outgroups, replacing missing linking score values by the score reported in the last they had provided, adjusting it by a decrement equivalent to 0.2 standard deviations derived from the variability in changing liking scores. Panel f (N = 13,702) depicts equivalent effects for perceived intelligence. These effects were estimated using linear regression models, controlling for participants' age, gender, household income, educational attainment, region of residence, and dummies for wave-specific treatment conditions. This figure considers as the grouping criterion the reported intentions to vote for Lula (lulistas) or Bolsonaro (bolsonaristas) in the first election round. To mitigate endogeneity concerns (i.e., participants changing both their political identification and affective polarization scores over time due to unobserved factors), we used as grouping criterion the participants' voting intentions reported in wave 2. Models exclude participants who did not report intentions to vote for either Lula or Bolsonaro and were estimated with clustered standard errors at the individual level. Data are presented as model-predicted mean values, with error bars indicating 95% confidence intervals. All statistical tests were two-sided.

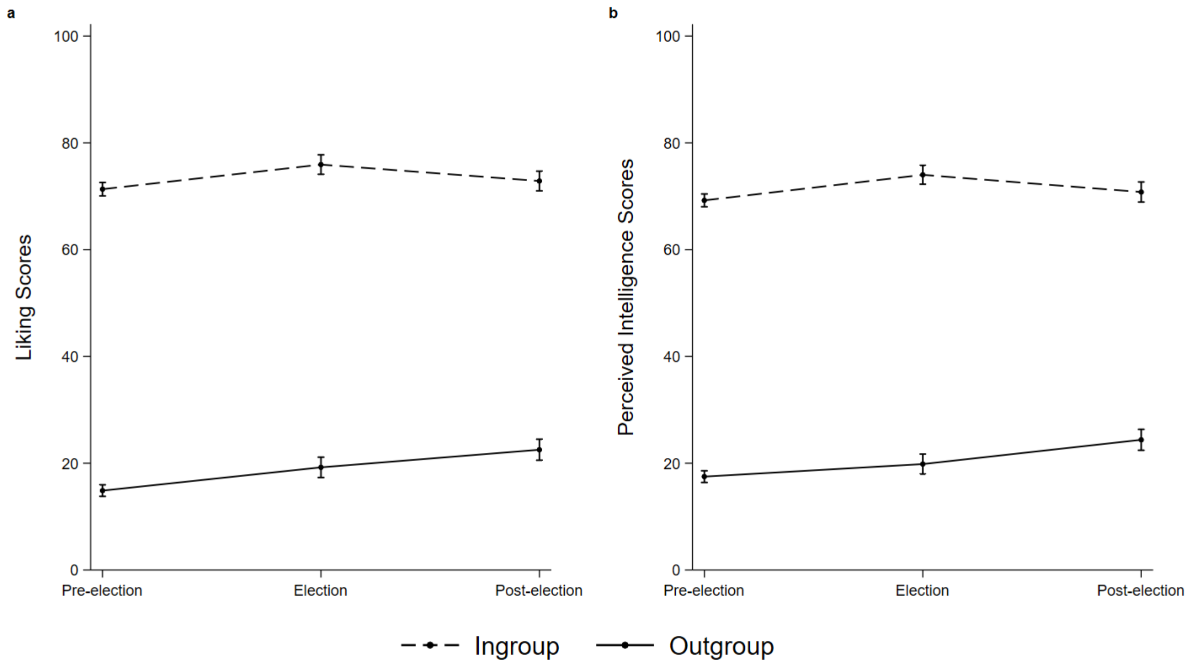

Supplementary Fig. 5 | Evolution of affective polarization for lulistas and bolsonaristas over waves considering only participants assigned to control conditions.

Panel a (N = 7,052) depicts how average liking scores fluctuate before, during, and after the election period for political ingroups (individuals who share the respondent's political self-categorization) and outgroups (individuals who hold a different political self-categorization) considering only participants assigned to control conditions. Panel b (N = 7,050) depicts equivalent effects for perceived intelligence. These effects were estimated using linear regression models, controlling for participants' age, gender, household income, educational attainment, region of residence, and dummies for wave-specific treatment conditions. This figure considers as the grouping criterion the reported intentions to vote for Lula (lulistas) or Bolsonaro (bolsonaristas) in the first election round. To mitigate endogeneity concerns (i.e., participants changing both their political identification and affective polarization scores over time due to unobserved factors), we used as grouping criteria the participants' voting intentions reported in wave 2. Models exclude participants who did not report intentions to vote for either Lula or Bolsonaro and were estimated with clustered standard errors at the individual level. Data are presented as model-predicted mean values, with error bars indicating 95% confidence intervals. All statistical tests were two-sided.

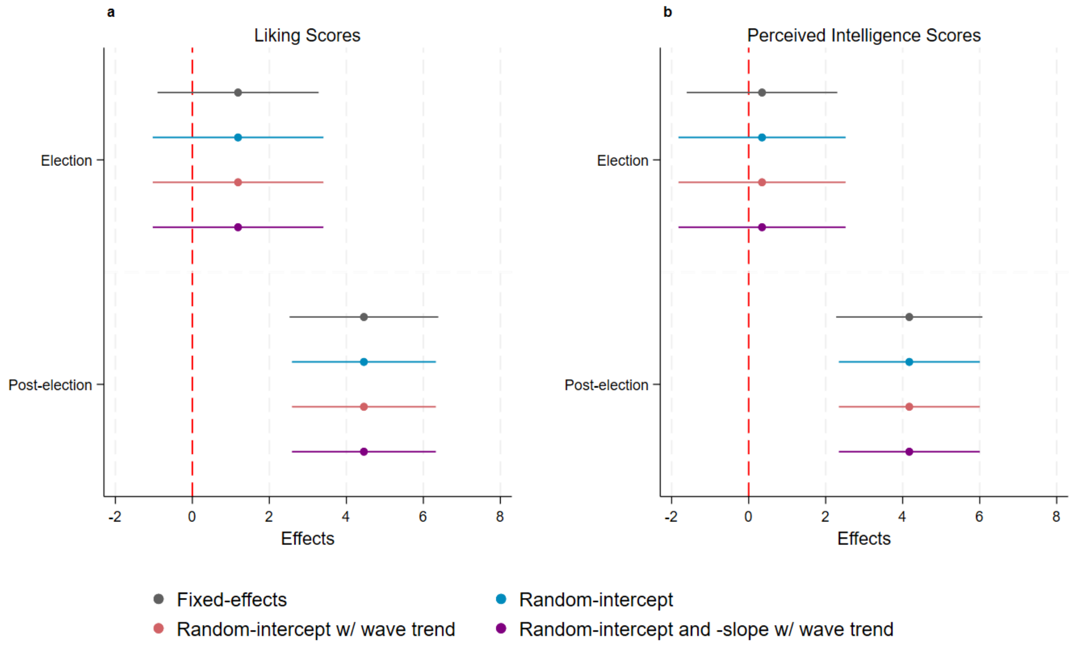

Supplementary Fig. 6 | Election effects across different model specifications.

Panel a (N = 13,594) depicts the change in average liking scores, compared to the pre-election, during and after the election period for political outgroups (individuals who hold a different political self-categorization). Panels b (N = 13,594) depicts equivalent effects for perceived intelligence. These effects were estimated using (a) linear regression models with clustered standard errors at the individual level, (b) mixed-effects models with random-intercept at the individual level, (c) mixed-effects models with random-intercept at the individual level and a linear wave trend term, and (d) mixed-effects models with two levels (wave and individual) with a linear wave trend term. This figure considers as the grouping criterion the reported intentions to vote for Lula (lulistas) or Bolsonaro (bolsonaristas) in the first election round. All models control for participants' age, sex, household income, education attainment, and region of residence, dummies for wave-specific treatment conditions. Data are presented as estimated mean changes, with error bars indicating 95% confidence intervals. All statistical tests were two-sided.

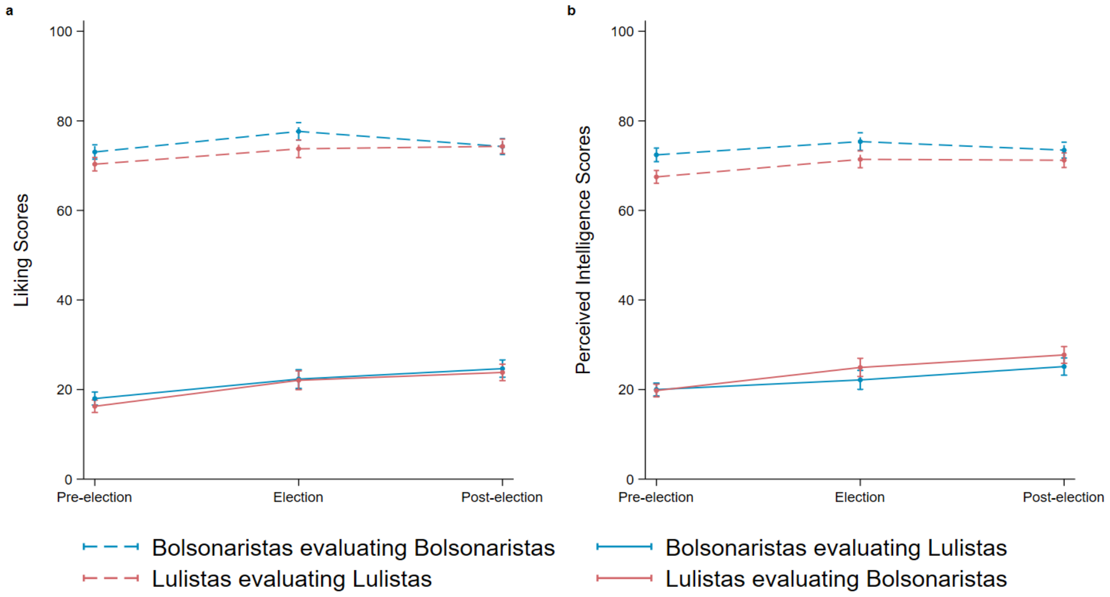

Supplementary Fig. 7 | Evolution of affective polarization for lulistas' and bolsonaristas' evaluations of ingroups and outgroups, using the intention-to-vote definition.

Panel a (N = 13,594) depicts the evolving trends in average liking scores, revealing how lulistas and bolsonaristas evaluate their ingroups (individuals who share the respondent's political self-categorization) and outgroups (individuals who hold a different political self-categorization) before, during, and after the election period. Panel b (N = 13,594) depicts equivalent effects for perceived intelligence. These effects were estimated using linear regression models, controlling for participants' age, sex, household income, education attainment, and region of residence, and dummies for wave-specific treatment conditions. This figure considers as the grouping criterion the reported intentions to vote for Lula (lulistas) or Bolsonaro (bolsonaristas) in the first election round. Models exclude participants who did not report intentions to vote for either Lula or Bolsonaro and were estimated with clustered standard errors at the individual level. Data are presented as model-predicted mean values, with error bars indicating 95% confidence intervals. All statistical tests were two-sided. Note: Results show that the losing group (bolsonaristas) had a steeper post-election reduction in ingroup evaluations particularly for liking (liking: $b = -3.93$, $t(2,581) = -3.14$, $p = 0.002$, 95% CI [-6.39, -1.48]; perceived intelligence: $b = -1.73$, $t(2,581) = -1.47$, $p = 0.143$, 95% CI [-4.04, 0.58]), and similar, rising gradient for outgroup evaluations relative to the winning group (liking: $b = 0.57$, $t(2,581) = 0.41$, $p = 0.681$, 95% CI [-2.15, 3.29]; perceived intelligence: $b = 0.19$, $t(2,581) = 0.13$, $p = 0.895$, 95% CI [-2.60, 2.97]).

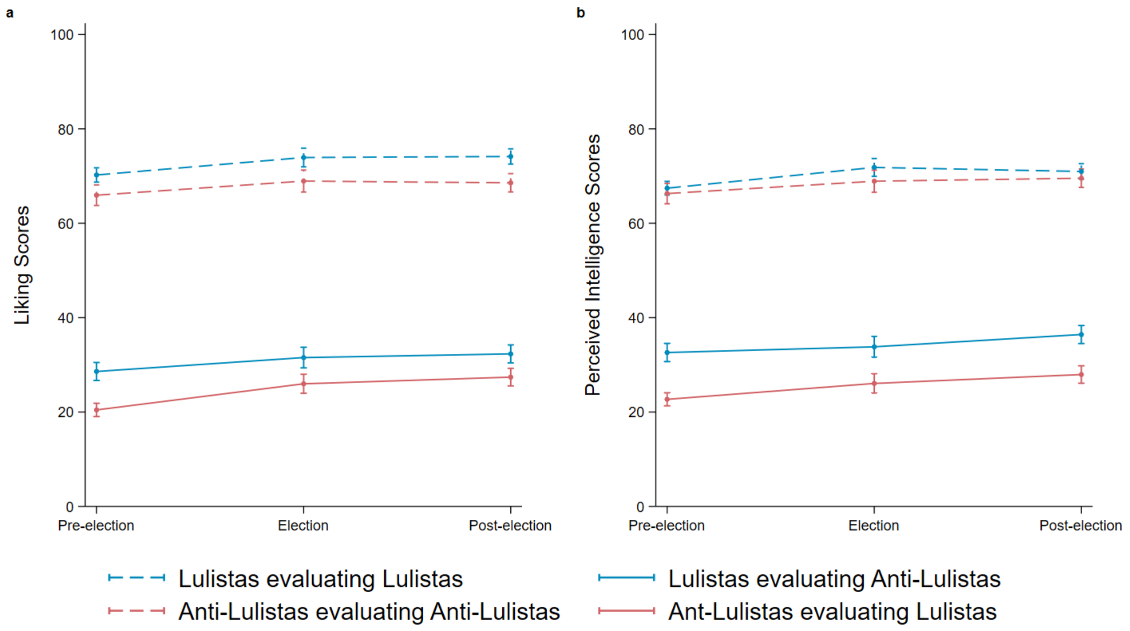

Supplementary Fig. 8 | Evolution of affective polarization for lulistas' and anti-lulistas' evaluations of ingroups and outgroups.

Panel a (N = 13,853) depicts the evolving trends in average liking scores, revealing how lulistas and anti-lulistas evaluate their ingroups (individuals who share the respondent's political self-categorization) and outgroups (individuals who hold a different political self-categorization) before, during, and after the election period. Panel b (N = 13,855) depicts equivalent effects for perceived intelligence. These effects were estimated using linear regression models, controlling for participants' age, sex, household income, education attainment, and region of residence, and dummies for wave-specific treatment conditions. This figure considers as grouping criteria the reported intentions to vote for Lula (lulistas) and those who stated they would never vote for Lula in the first election round. Models exclude participants who did not report intentions to vote for Lula or did not express anti-sentiment towards Lula and were estimated with clustered standard errors at the individual level. Data are presented as model-predicted mean values, with error bars indicating 95% confidence intervals. All statistical tests were two-sided.

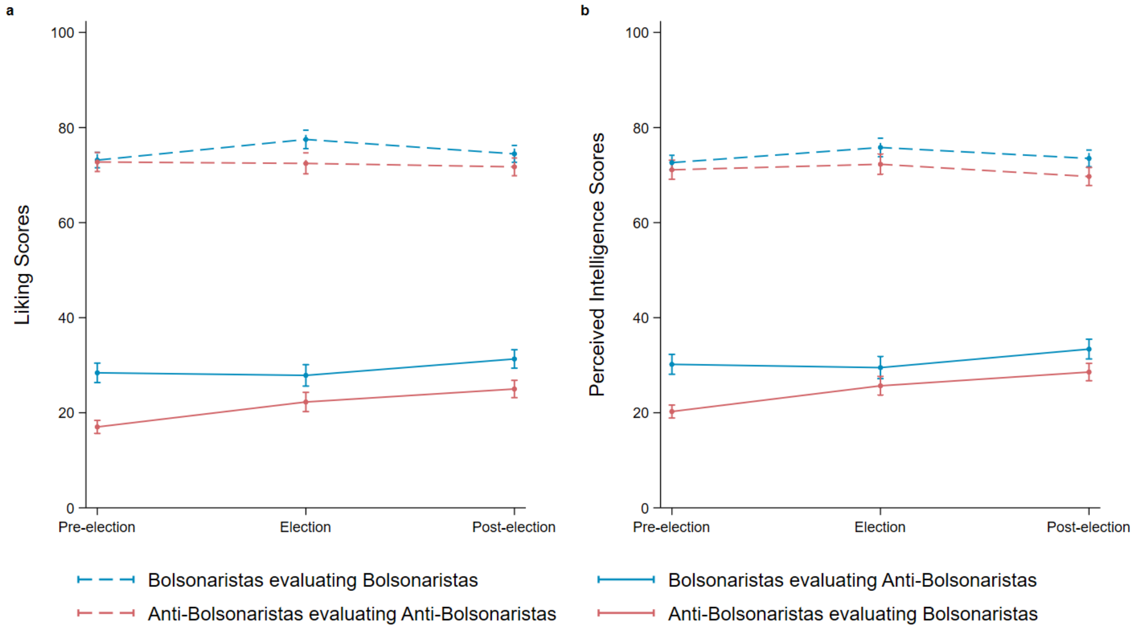

Supplementary Fig. 9 | Evolution of affective polarization for bolsonaristas' and anti-bolsonaristas' evaluations of ingroups and outgroups.

Panel a (N = 13,300) depicts the evolving trends in average liking scores, revealing how bolsonaristas and anti-bolsonaristas evaluate their ingroups (individuals who share the respondent's political self-categorization) and outgroups (individuals who hold a different political self-categorization) before, during, and after the election period. Panel b (13,300) depicts equivalent effects for perceived intelligence. These effects were estimated using linear regression models, controlling for participants' age, sex, household income, education attainment, and region of residence, and dummies for wave-specific treatment conditions. This figure considers as grouping criteria the reported intentions to vote for Bolsonaro (bolsonaristas) and those who stated they would never vote for Bolsonaro in the first election round. Models exclude participants who did not report intentions to vote for Bolsonaro or did not express anti-sentiment towards either Bolsonaro and were estimated with clustered standard errors at the individual level. Data are presented as model-predicted mean values, with error bars indicating 95% confidence intervals. All statistical tests were two-sided.

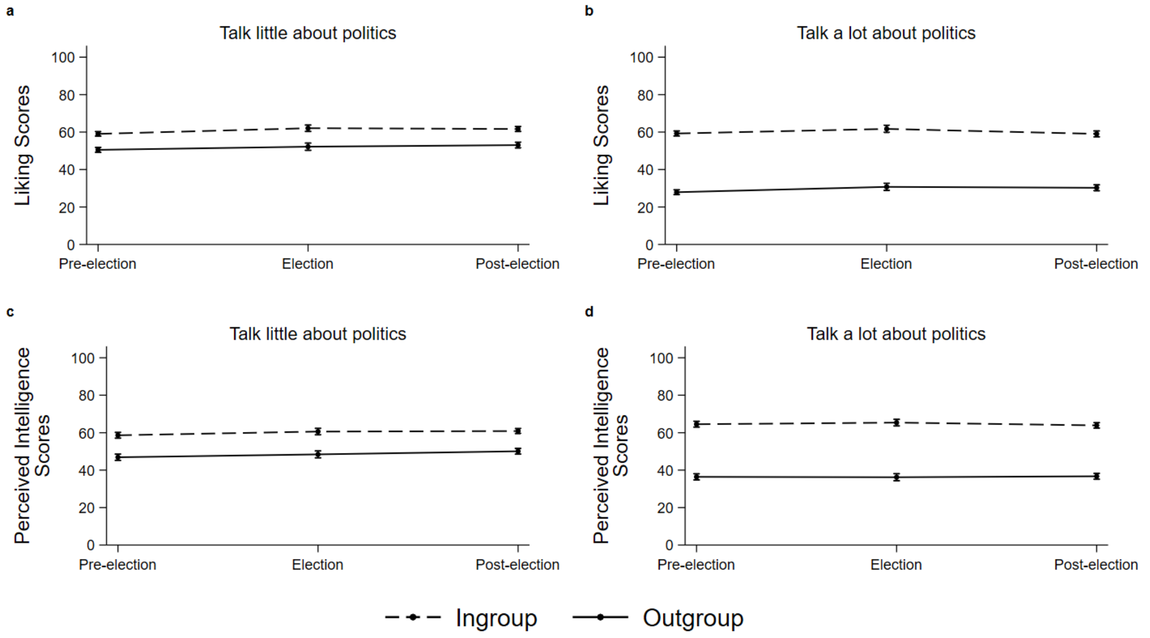

Supplementary Fig. 10 | Evolution of affective polarization for self-identified left-wing and right-wing considering evaluated targets that talk little and a lot about politics.

Panel a (N = 11,244) depicts trends in average liking scores, revealing how left- and right-wing respondents evaluate their ingroups (individuals who share the respondent's political self-categorization) and outgroups (individuals who hold a different political self-categorization) who talk little about politics before, during, and after the election period. Panel b (N = 11,244) depicts equivalent effects for perceived intelligence. Panel c (N = 9,332) depicts trends in average liking scores, revealing how left- and right-wing respondents evaluate their ingroups and outgroups who talk a lot about politics before, during, and after the election period. Panel d (N = 9,332) depicts equivalent effects for perceived intelligence. These effects were estimated using linear regression models, controlling for participants' age, gender, household income, educational attainment, region of residence, and dummies for wave-specific treatment conditions. This figure considers as the grouping criterion the reported ideological left-right self-identification. To mitigate endogeneity concerns (i.e., participants changing both their political identification and affective polarization scores over time due to unobserved factors), we used as grouping criteria the participants' ideological identification reported in wave 2. Models exclude participants who reported a "center" ideology, refused to identify as left, center, or right, or who lacked knowledge about the meaning of these ideological labels, and were estimated with clustered standard errors at the individual level. Data are presented as model-predicted mean values, with error bars indicating 95% confidence intervals. All statistical tests were two-sided.

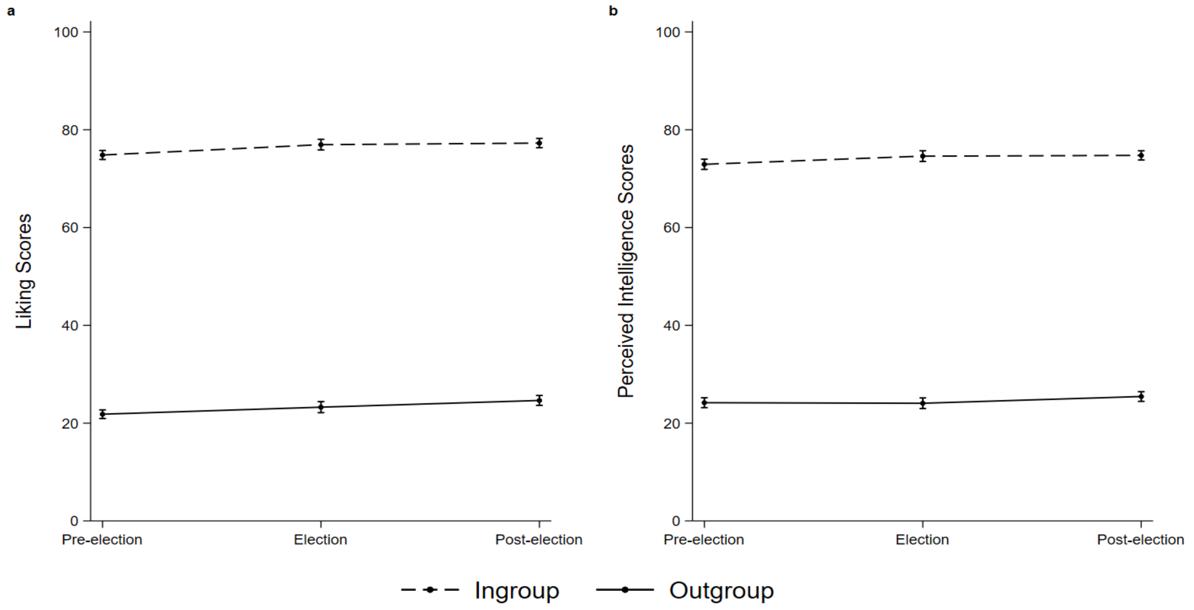

Supplementary Fig. 11 | Evolution of meta-polarization over waves.

Panel a (N = 19,758) depicts how average meta-liking scores fluctuate before, during, and after the election period for political ingroups (individuals who share the respondent's political self-categorization) and outgroups (individuals who hold a different political self-categorization). Panel b (N = 17,814) depicts equivalent effects for meta-perceived intelligence. These effects were estimated using linear regression models, controlling for participants' age, gender, household income, educational attainment, region of residence, and dummies for wave-specific treatment conditions. This figure considers as the grouping criterion the reported intentions to vote for Lula (lulistas) or Bolsonaro (bolsonaristas) in the first election round. To mitigate endogeneity concerns (i.e., participants changing both their political identification and affective polarization scores over time due to unobserved factors), we used as the grouping criterion the participants' voting intentions reported in wave 2. Models exclude participants who did not report intentions to vote for either Lula or Bolsonaro and were estimated with clustered standard errors at the individual level. Data are presented as model-predicted mean values, with error bars indicating 95% confidence intervals. All statistical tests were two-sided. Compared to the pre-election period, we did not find evidence that affective polarization, as defined by liking scores, changed during (b = 0.68, $t(3{,}523) = 0.82$, $p = 0.411$, 95% CI [-0.94, 2.29], standardized coefficient = 0.004) or after the elections (b = -0.38, $t(3{,}523) = -0.42$, $p = 0.634$, 95% CI [-1.95, 1.19], standardized coefficient = -0.003). These effects were statistically equivalent ($t(3{,}523) = 3.89$, $p < 0.001$). When considering intelligence scores, we observed a significant increase in meta-polarization during (vs. before) the elections (b = 1.79, $t(2{,}923) = 2.17$, $p = 0.030$, 95% CI [0.17, 3.40], standardized coefficient = 0.01), whereas in the subsequent period there was no significant difference (b = 0.57, $t(2{,}923) = 0.69$, $p = 0.490$, 95% CI [-1.04, 2.17], standardized coefficient = .004). This latter non-significant effect fell within the upper and lower equivalence bounds of the SESOI ($t(2{,}923) = 3.17$, $p < 0.001$).

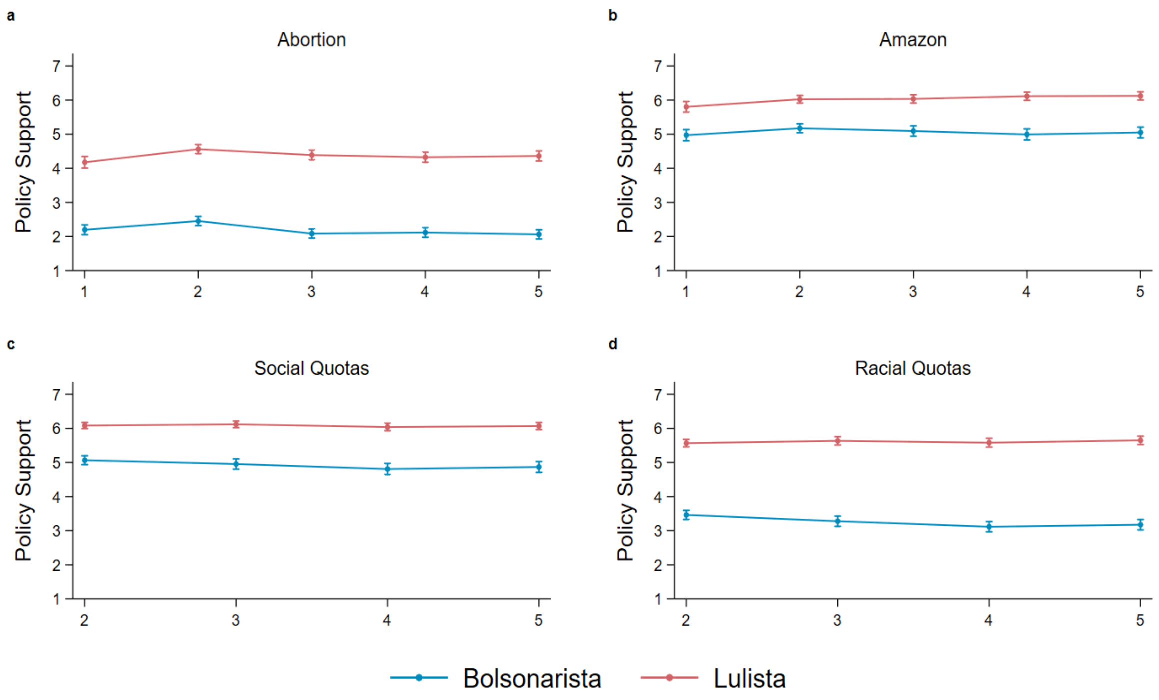

Supplementary Fig. 12 | Evolution of policy support.

Panel a (N = 13,608) depicts how average support for the legalization of abortion fluctuate over the five-wave period for lulistas and bolsonaristas. Panel b (N = 13,608), c (N = 11,172), and d (N = 11,172) depict equivalent effects for support for zero deforestation in the Amazon, access to universities through social quotas, and racial quotas, respectively. These effects were estimated using linear regression models, controlling for participants' age, sex, household income, education attainment, and region of residence. This figure considers as grouping criteria the reported intentions to vote for Lula (lulistas) or Bolsonaro (bolsonaristas) in the first election round. To mitigate endogeneity concerns (i.e., participants changing both their political identification and affective polarization scores over time due to unobserved factors), we used as grouping criteria the participants' voting intentions reported in wave 2. Models exclude participants who did not report intentions to vote for either Lula or Bolsonaro and were estimated with clustered standard errors at the individual level. Data are presented as model-predicted mean values, with error bars indicating 95% confidence intervals. All statistical tests were two-sided.

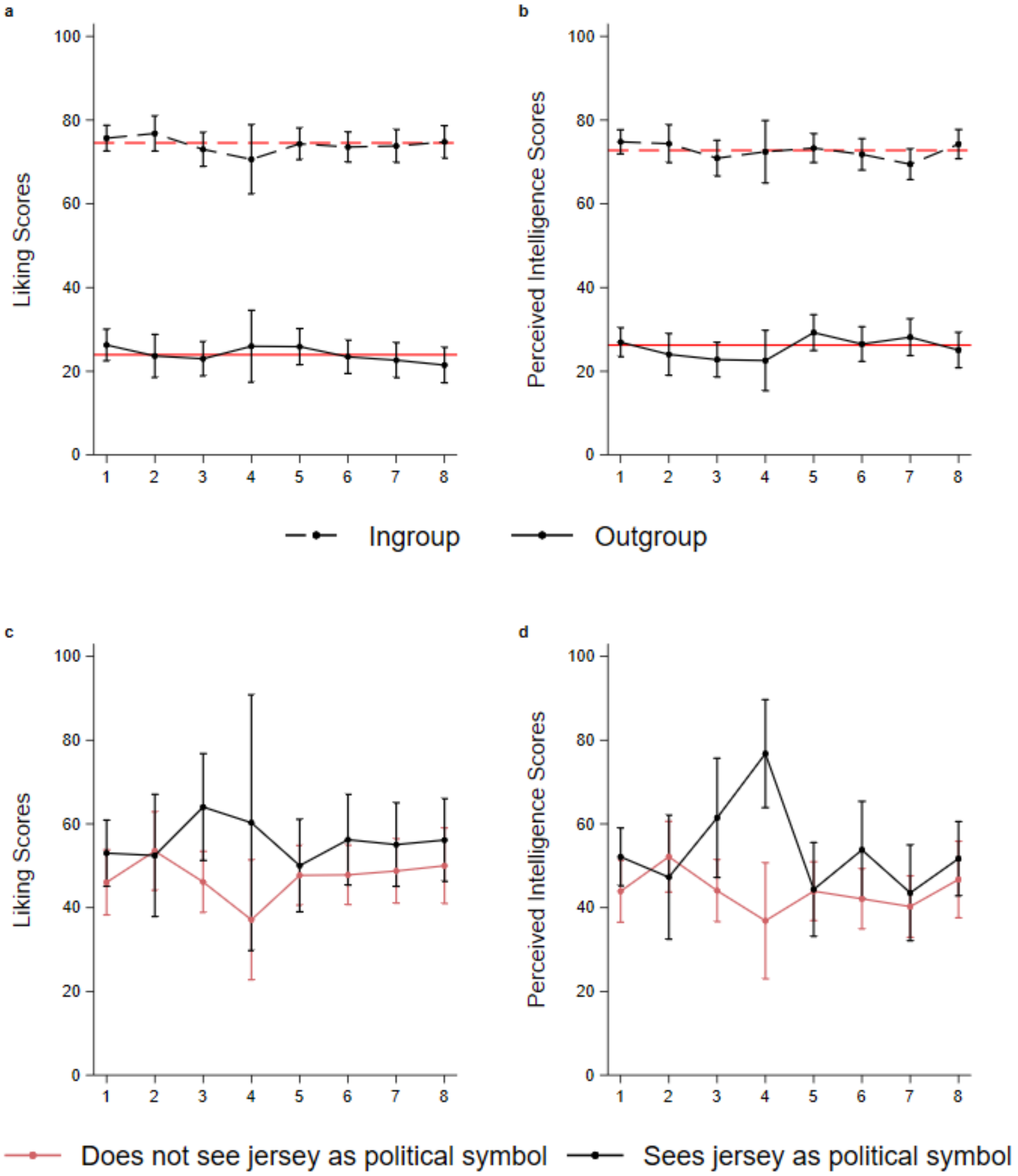

Supplementary Fig. 13 | Evolution of affective polarization during the World Cup.

Panel a (N = 2,334) depicts how average liking scores fluctuate during the World Cup for political ingroups (individuals who share the respondent's political self-categorization) and outgroups (individuals who hold a different political self-categorization). Panel b (N = 2,332) depicts equivalent effects for perceived intelligence. Panels c (N = 2,328) and d (N = 2,328) depict how average liking and perceived intelligence scores, respectively, fluctuated during the World Cup for those who perceive the Brazilian football shirt as a symbol of within-country political groups, and those who do not. These effects were estimated using linear regression

models, controlling for participants' age, sex, household income, educational attainment, region of residence, and dummies for wave-specific treatment conditions. This figure considers as the grouping criterion the reported intentions to vote for Lula (lulistas) or Bolsonaro (bolsonaristas) in the first election round. To mitigate endogeneity concerns (i.e., participants changing both their political identification and affective polarization scores over time due to unobserved factors), we used as the grouping criterion the participants' voting intentions reported in wave 2. Models exclude participants who did not report intentions to vote for either Lula or Bolsonaro and were estimated with clustered standard errors at the individual level. The red dashed and solid lines in panels a and b represent the average liking (or perceived intelligence) scores in all waves for political ingroups and outgroups, respectively. Data are presented as model-predicted mean values, with error bars indicating 95% confidence intervals. All statistical tests were two-sided.

Post-election events:

Supplementary Note 1: The World Cup and storming of federal government buildings

For many nationalities, and Brazil in particular, the FIFA World Cup may be a period of increased overall unity and strengthened national identity. In theory, intergroup disparities may recede to give place to collective solidarity, pride, and a simple common goal: support the national team. The fourth wave of data collection, in November-December 2022, assessed the extent to which this independent event could influence, and arguably, attenuate affective polarization. The wave was divided into eight sub-waves with the first just prior to Brazil's first match of the World Cup, and the remaining sub-waves after each of Brazil's subsequent matches (and after the matches that Brazil would have played, had the team not been eliminated from the competition in the quarter finals). The study was pre-registered in aspredicted.org (pre-registration #114066: https://aspredicted.org/c8xm2.pdf), with the following hypotheses:

H1: affective polarization in Brazil will be reduced while Brazil is competing in the FIFA World Cup 2022.
H1a: the effect advanced in H1 will be stronger after Brazil wins a match compared to not winning.
H2: meta-polarization in Brazil will be reduced while Brazil is competing in the FIFA World Cup 2022.
H2a: the effect advanced in H2 will be stronger after Brazil wins a match compared to not winning.

Overall, we found evidence that affective polarization was not associated with Brazil's performance (i.e. wins and losses), except for perceived intelligence after defeats, for which results were neither statistically significant nor statistically equivalent. Affective polarization remained steady and strong. Comparing wave 4 (the average of these sub-waves) to wave 3 (the period between the first election round and the runoff), we found evidence for a small reduction in affective polarization (liking: b = -2.95, $t(2,581) = -2.39$, $p = 0.017$, 95% CI [-5.38, -0.53]; perceived intelligence: b = -2.99, $t(2,581) = -2.59$, $p = 0.010$, 95% CI [-5.25, -0.72]), noting that this wave of the post-election period is perfectly confounded with the World Cup's timing.

Since Bolsonaro started his campaign for the presidency, for some people, the Brazilian national football shirt has become a political symbol, and is associated with the Bolsonarismo. We, thus, further assessed in an exploratory analysis, separate trends across sub-waves for those who ascribed this within-country group-based political meaning to the team shirt (N = 622) versus those who, for them, said that it only symbolized Brazil as a whole (N = 1,266, Supplementary Fig. 13, panels c and d). This analysis suggests that around the 4th sub-wave, when Brazil experienced a shock loss to Cameroon, those who perceived the shirt as a political

symbol experienced a non-significant rise in the liking measure of affective polarization (b = 7.27, SE = 16.07, p = 0.651) and a significant rise in the perceived intelligence measure (b = 24.66, SE = 7.45, p = 0.001), while those who did not see the team shirt as such had non-significant decreases in affective polarization for both measures (liking: b = -8.94, SE = 8.29, p = 0.281 liking; perceived intelligence: b = -7.05, SE = 8.00, p = 0.378), though these results should be treated with caution due to the small sample sizes ($N_{subwave\_4}$ = 70, $N_{other\ subwaves}$ = 267 on average). Similar results are obtained for alternative political groupings: left-right self-placement or anti-lulista versus anti-bolsonarista sentiment (see Supplementary Fig.15 and 16, respectively).

On January 8, 2023, after Bolsonaro's defeat and Lula's inauguration, rioters invaded the National Congress, the Supreme Court, and the Presidential Palace in protest against the results of the presidential election. Our longitudinal design allowed us to also evaluate potential changes in affective polarization related to these events. Unsurprisingly, the events sparked disparate reactions among lulistas and bolsonaristas in how they evaluated the reasons for the riots (32.2% of bolsonaristas vs. 9.65% of lulistas agreed with the reasons for the riots, as they perceived them) and even in how they characterized them (28.32% of bolsonaristas vs. 82.46% of lulistas as a terrorist attack; 48.64% of bolsonaristas vs. 8.05% of lulistas considered them to be a legitimate protest). Given these differing reactions, one might expect an intensification of affective polarization in the weeks afterwards. However, as noted in the main manuscript, affective polarization remained remarkably stable: we find no evidence of changes in polarization in liking (b = -0.61, SE = 1.18, p = 0.604) or perceived intelligence (b = -1.62, SE = 1.11, p = 0.146) between the World Cup wave (pre-January 8th) and the last wave (late-January to February), as confirmed by bayes factors and equivalence tests (see Supplementary Table 5).

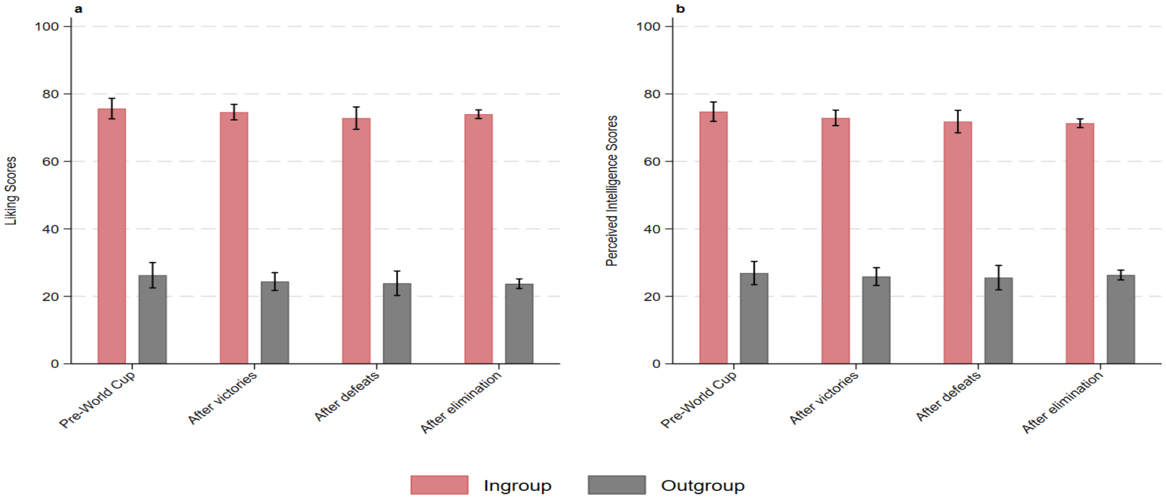

Supplementary Fig. 14 | Effect of Brazil team's performance on affective polarization during the World Cup.

Panels a (N = 4,840) depicts average liking scores before the World Cup, following Brazil's victories and defeats, and after Brazil's elimination from the tournament for political ingroups and outgroups. Panel b (N = 4,840) depicts equivalent effects for perceived intelligence. These effects were estimated using linear regression models, controlling for participants' age, gender, household income, educational attainment, and region of residence. This figure considers as grouping criteria the reported intentions to vote for Lula or Bolsonaro in the first election round. To mitigate endogeneity concerns (i.e., participants changing both their political identification and affective polarization scores over time due to unobserved factors), we used as grouping criteria the participants' voting intentions reported in wave 2. Models exclude participants who did not report intentions to vote for either Lula or Bolsonaro and were estimated with clustered standard errors at the individual level. Error bars indicate 95% confidence intervals. Compared to the first sub-wave, we did not find evidence that affective polarization changed systematically during the World Cup (liking: b = 1.21, SE = 3.11, $p = 0.698$; perceived intelligence: b = -1.59, SE = 2.89, $p = 0.582$). The coefficients for liking and intelligence are not only non-significant, but also in opposite directions. A TOST procedure suggests these are not meaningful effects (liking: $p = 0.061$; perceived intelligence: $p = 0.09$). Considering the performance of the Brazilian national team, there was no evidence that affective polarization differed after victories (liking: b = 0.84, SE = 3.43, $p = 0.808$; perceived intelligence: b = -0.83, SE = 3.24, $p = 0.799$) or defeats (liking: b = 0.71, SE = 2.88, $p = 0.806$; perceived intelligence: b = -2.72, SE = 2.65, $p = 0.304$). Importantly, in most cases the TOST corroborates the null. Whether the Brazilian team won (liking: $p = 0.012$; perceived intelligence: $p = 0.015$) or lost (liking: $p = 0.05$; perceived intelligence: $p = 0.093$), affective polarization was not overall affected by the performance of the Brazilian team (except for perceived intelligence after defeats, for which results are inconclusive, i.e. neither statistically significant nor statistically equivalent).

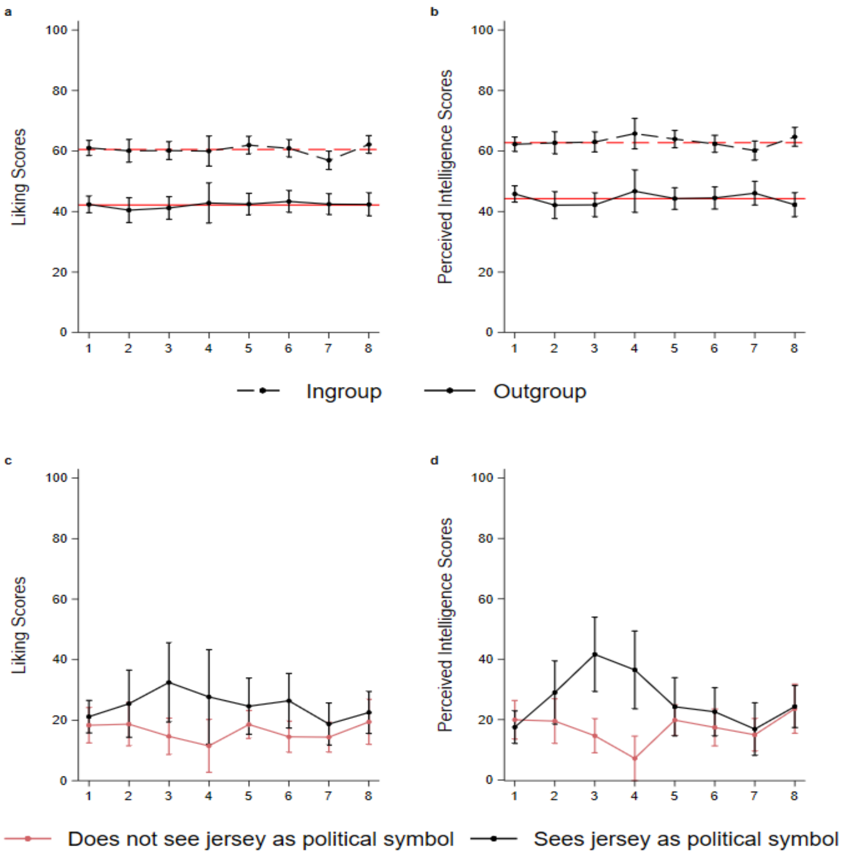

Supplementary Fig. 15 | Evolution of affective polarization during the World Cup considering self-identified left-wing and right-wing respondents.

Panel a (N = 1,956) depicts how average liking scores fluctuate during the World Cup for political ingroups (individuals who share the respondent's political self-categorization) and outgroups (individuals who hold a different political self-categorization). Panel b (N = 1,956) depicts equivalent effects for perceived intelligence. Panels c (N = 1,778) and d (N = 1,778) depict how average liking and perceived intelligence scores, respectively, fluctuated during the World Cup for those who perceive the Brazilian football shirt as a symbol of within-country political groups, and those who do not. These effects were estimated using linear regression models, controlling for participants' age, sex, household income, educational attainment, region of residence, and dummies for wave-specific treatment conditions. This figure considers as grouping criteria the reported ideological left-right self-identification. To mitigate endogeneity concerns (i.e., participants changing both their political identification and affective polarization scores over time due to unobserved factors), we used as grouping criteria the participants' ideological identification reported in wave 2. Models exclude participants who reported a "center" ideology, refused to identify as left, center, or right, or who lacked knowledge about the meaning of these ideological labels and were estimated with clustered standard errors at the individual level. The red dashed and solid lines in panels a and b represent the average liking (or perceived intelligence) scores in all waves for political ingroups and outgroups, respectively. Data are presented as model-predicted mean values, with error bars indicating 95% confidence intervals. All statistical tests were two-sided.

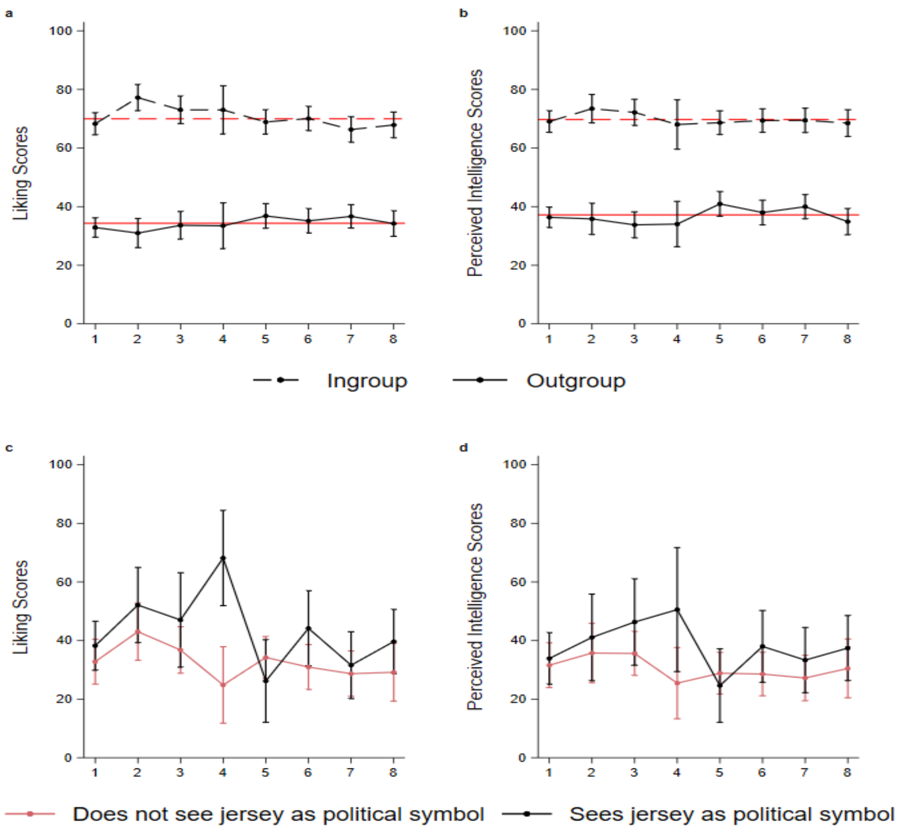

Supplementary Fig. 16 | Evolution of affective polarization during the World Cup considering anti-Lula and anti-Bolsonaro respondents.

Panel a (N = 2,750) depicts how average liking scores fluctuate during the World Cup for political ingroups (individuals who share the respondent's political self-categorization) and outgroups (individuals who hold a different political self-categorization). Panel b (N = 2,748) depicts equivalent effects for perceived intelligence. Panels c (N = 2,744) and d (N = 2,744) depict how average liking and perceived intelligence scores, respectively, fluctuated during the World Cup for those who perceive the Brazilian football shirt as a symbol of within-country political groups, and those who do not. These effects were estimated using linear regression models, controlling for participants' age, gender, household income, educational attainment, region of residence, and dummies for wave-specific treatment conditions. This figure considers as grouping criteria the reported anti-Lula or anti-Bolsonaro sentiment. To mitigate endogeneity concerns (i.e., participants changing both their political identification and affective polarization scores over time due to unobserved factors), we used as grouping criteria the participants' anti-politician sentiment reported in wave 2. Panels a and b exclude participants who either did not express anti-sentiment towards either Lula or Bolsonaro or who reported anti-sentiment towards both figures and were estimated with clustered standard errors at the individual level. The red dashed and solid lines in panels a and b represent the average liking (or perceived intelligence) scores in all waves for political ingroups and outgroups, respectively. Data are presented as model-predicted mean values, with error bars indicating 95% confidence intervals. All statistical tests were two-sided.

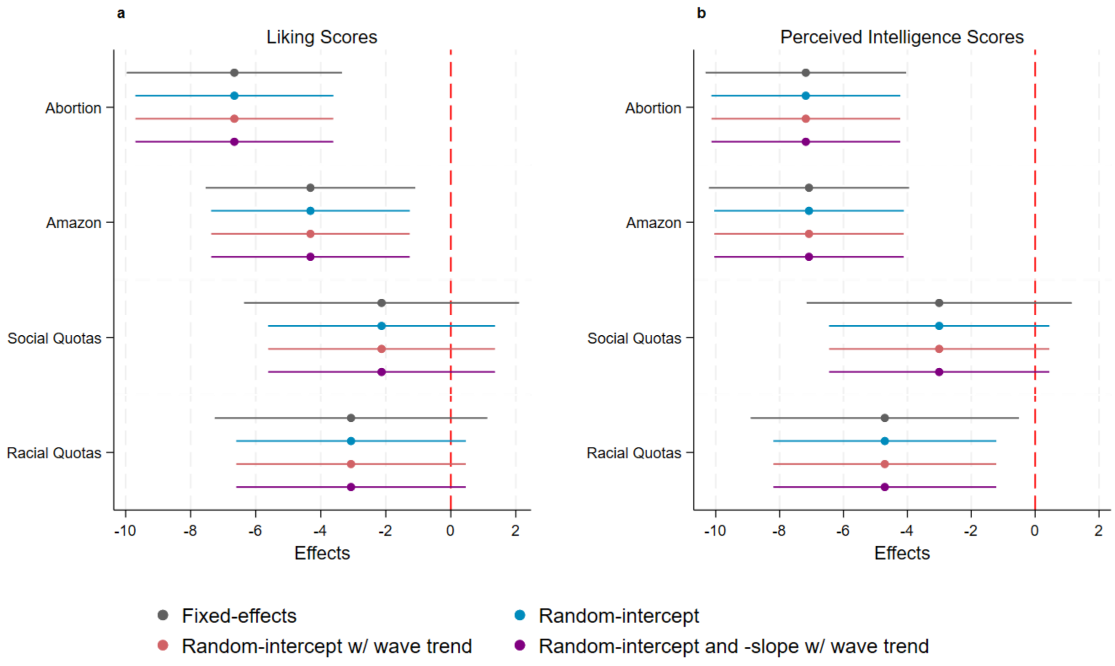

Supplementary Fig. 17 | Treatment effects across different model specifications.

Panel a (N = 6,530) depicts average effects of misperception-correcting information across experimental conditions on liking scores for political outgroups (individuals who hold a different political self-categorization). Panels b (N = 6,530) depicts equivalent effects for perceived intelligence. These effects were estimated using (a) linear regression models with clustered standard errors at the individual level, (b) mixed-effects models with random-intercept at the individual level, (c) mixed-effects models with random-intercept at the individual level and a linear wave trend term, and (d) mixed-effects models with two levels (wave and individual) with a linear wave trend term. This figure considers as the grouping criterion the reported intentions to vote for Lula (lulistas) or Bolsonaro (bolsonaristas) in the first election round. Data are presented as estimated mean changes, with error bars indicating 95% confidence intervals. All statistical tests were two-sided.

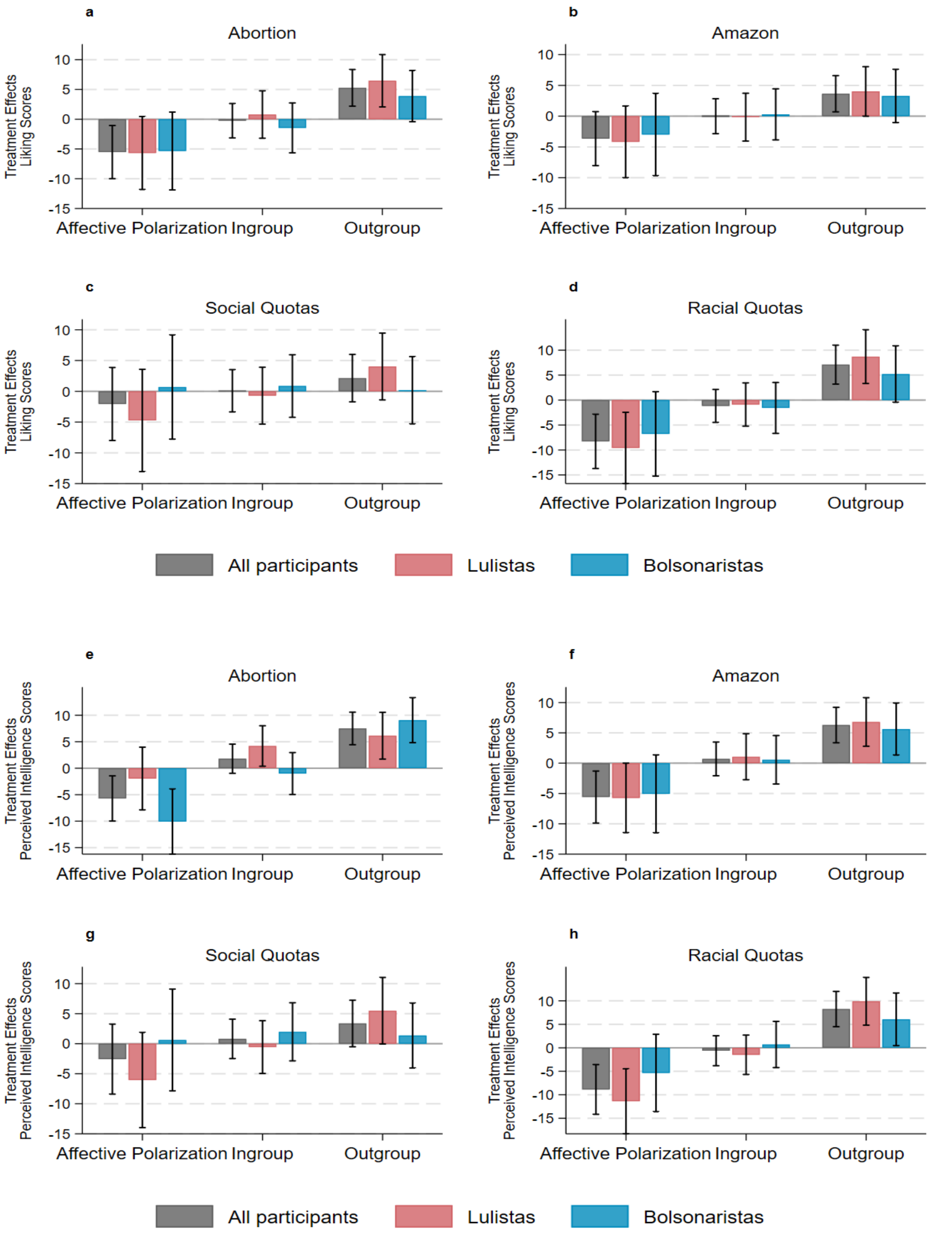

Supplementary Fig. 18 | The effect of providing misperception-correcting information for the ingroups and outgroups of lulistas and bolsonaristas in waves 2 and 4.

Panels a (N = 3,672) and e (N = 3,672) depict the average effect of the abortion treatment on liking and perceived intelligence scores, respectively, for political ingroups (individuals who share the respondent's political self-categorization), outgroups (individuals who hold a different political self-categorization), and their difference (affective polarization) in wave 2. Panels b (N = 3,672) and f (N = 3,672) show the effect of the Amazon treatment in the same wave 2. Panels c (N = 2,360) and g (N = 2,358) depict the social quotas treatment effect in wave 4, and panels d (N = 2,360) and h (N = 2,358) replicate this analysis for the racial quotas treatment effect. This figure considers as grouping criterion reported intentions to vote for Lula (lulistas) or Bolsonaro (bolsonaristas) in the first election round. These effects were estimated using linear regression models with fixed-effects and clustered standard errors at the individual level. Data are presented as estimated mean changes, with error bars indicating 95% confidence intervals. All statistical tests were two-sided.

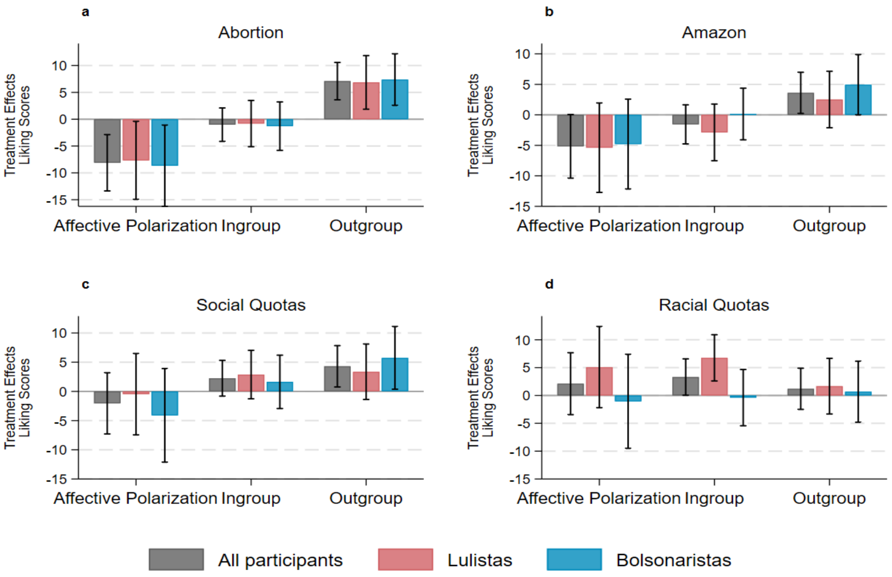

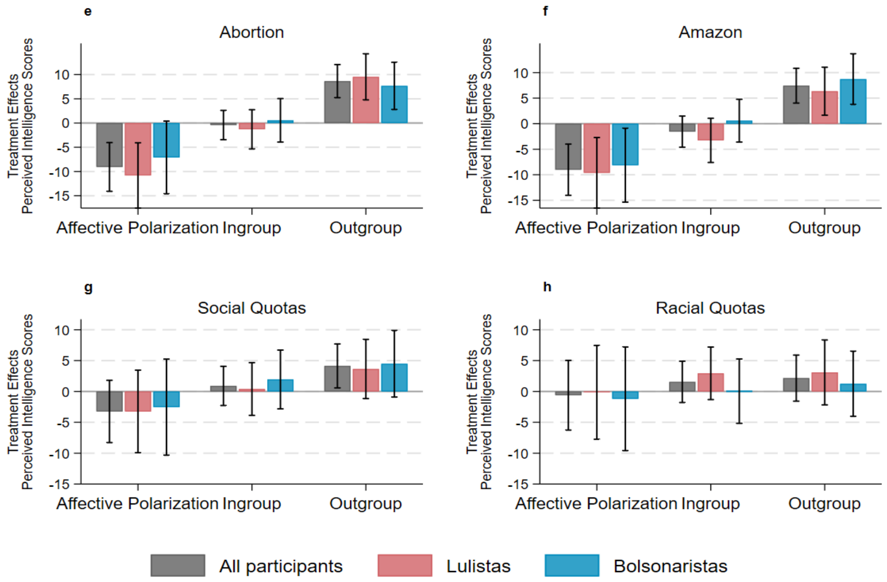

Supplementary Fig. 19 | The effect of misperception-correcting information for the ingroups and outgroups of lulistas and bolsonaristas in waves 3 and 5.

Panels a (N = 2,858) and e (N = 2,858) depict the average effect of the abortion treatment on liking and perceived intelligence scores, respectively, for political ingroups (individuals who share the respondent's political self-categorization), outgroups (individuals who hold a different political self-categorization), and their difference (affective polarization) in wave 3. Panels b (N = 2,858) and f (N = 2,858) show the effect of the Amazon treatment in the same wave 3. Panels c (N = 2,552) and g (N = 2,554) depict the social quotas treatment effect in wave 5, and panels d (N = 2,552) and h (N = 2,554) replicate this analysis for the racial quotas treatment effect. This figure considers as grouping criterion reported intentions to vote for Lula (lulistas) or Bolsonaro (bolsonaristas) in the first election round. These effects were estimated using linear regression models with fixed-effects and clustered standard errors at the individual level. Data are presented as estimated mean changes, with error bars indicating 95% confidence intervals. All statistical tests were two-sided.

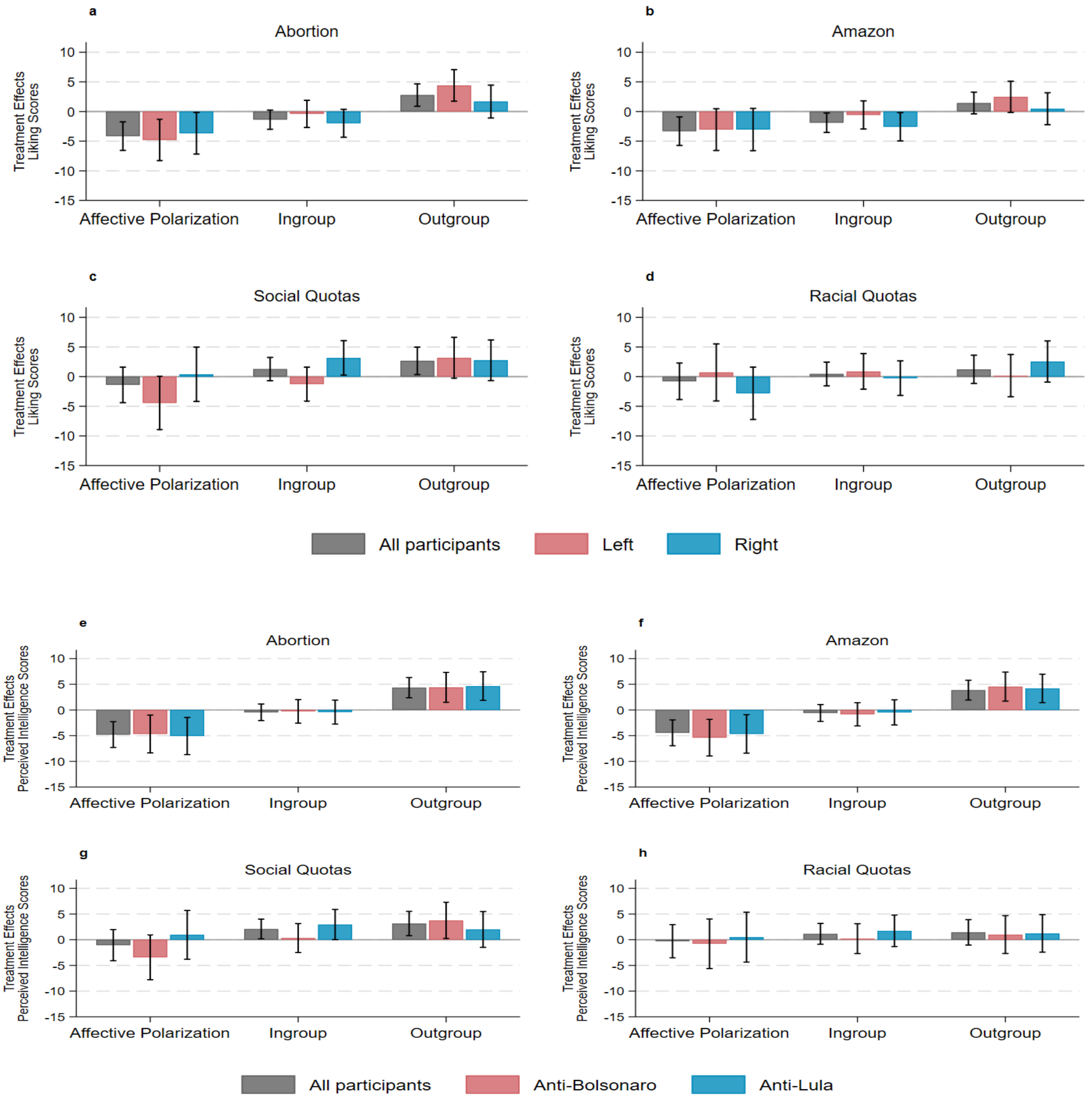

Supplementary Fig. 20 | The effect of providing misperception-correcting information on liking and perceived intelligence scores for the ingroups and outgroups of self-identified left-wing and right-wing individuals.

Panel a (N = 5,444) depicts the average effect of misperception-correcting information about support for legalizing abortion in the first 3 months of pregnancy on liking scores for political ingroups (individuals who share the respondent's political self-categorization), outgroups (individuals who hold a different political self-categorization), and their difference (affective polarization). Panels b (N = 5,444), c (N = 4,134), and d (N = 4,136) depict equivalent effects of misperception-correcting information about support for zero deforestation in the Amazon, access to universities through social and racial quotas, respectively. Panels e-h replicate these effects for the perceived intelligence scores. This figure considers as grouping criterion the ideological left-right self-identification. These effects were estimated using linear regression models with fixed-effects and clustered standard errors at the individual level. Data are presented as estimated mean changes, with error bars indicating 95% confidence intervals. All statistical tests were two-sided.

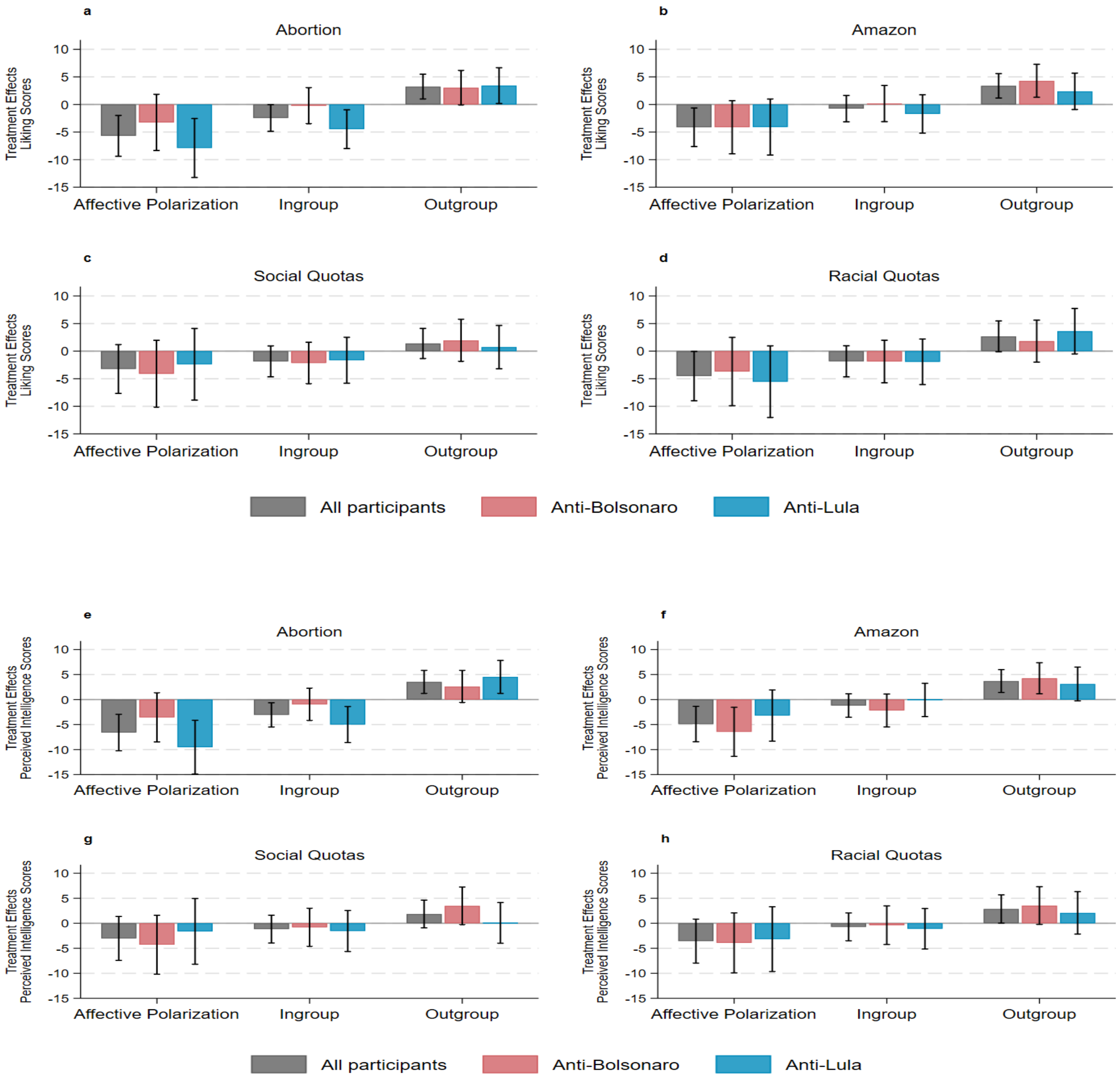

Supplementary Fig. 21 | The effect of providing misperception-correcting information on liking and perceived intelligence scores for the ingroups and outgroups of people holding anti-Lula or anti-Bolsonaro sentiment.

Panel a (N = 7,764) depicts the average effect of misperception-correcting information about support for legalizing abortion in the first 3 months of pregnancy on liking scores for political ingroups (individuals who share the respondent's political self-categorization), outgroups (individuals who hold a different political self-categorization), and their difference (affective polarization). Panels b (N = 7,764), c (N = 5,826), and d (N = 5,828) depict equivalent effects of misperception-correcting information about support for zero deforestation in the Amazon, access to universities through social and racial quotas, respectively. Panels e-h replicate these effects for the perceived intelligence scores. This figure considers as the grouping criterion those who stated they would never vote for Lula/ Bolsonaro in the first-round of the elections. These effects were estimated using linear regression models with fixed-effects and clustered standard errors at the individual level. Data are presented as estimated mean changes, with error bars indicating 95% confidence intervals. All statistical tests were two-sided.

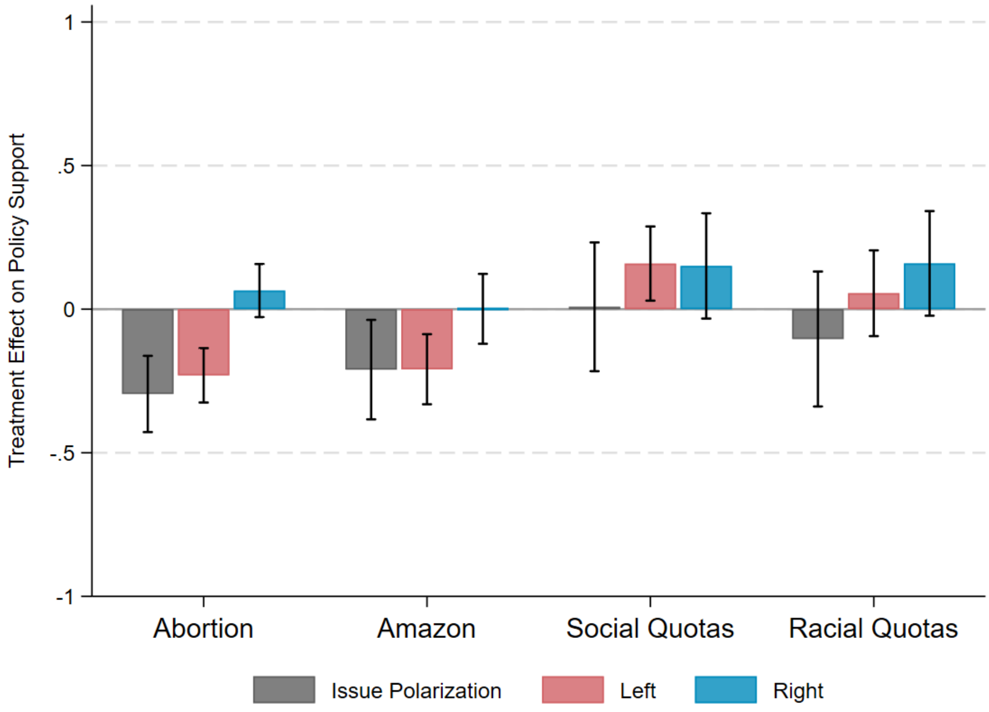

Supplementary Fig. 22 | The effect of providing misperception-correcting information on policy support for self-identified left-wing and right-wing individuals.

This figure depicts changes in participants' policy support towards abortion within the first three months of pregnancy (N = 4,234), zero deforestation (N = 4,232), social quotas (N = 2,977), and racial quotas (N = 2,959) among self-identified left-wing or self-identified right-wing respondents, and their difference in support (issue polarization), after versus before receiving misperception-correcting information about the respective policy. These effects were estimated using linear regression models controlling for age, sex, education, income and region, with clustered standard errors at the individual level. Data are presented as estimated mean changes, with error bars indicating 95% confidence intervals. All statistical tests were two-sided. Even though both the abortion and amazon treatments significantly reduced polarization over the respective policies, we did not find evidence that either the social quotas or the racial quotas treatments affected issue polarization (social quotas: b = 0.008, $t(1{,}490)$ = 0.07, p = 0.943, 95% CI [-0.21, 0.23], standardized coefficient = 0.001; racial quotas: b = -0.10, $t(1{,}490)$ = -0.87, p = 0.386, 95% CI [-0.34, 0.13], standardized coefficient = -0.01).

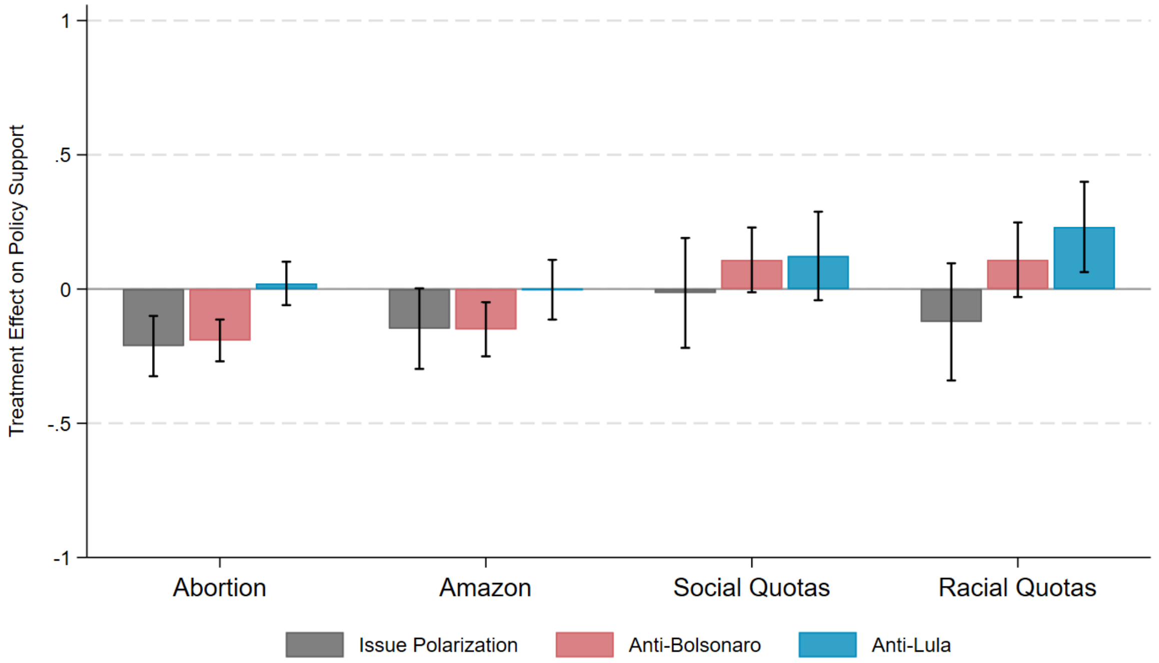

Supplementary Fig. 23 | The effect of providing misperception-correcting information on policy support for people holding anti-Lula or anti-Bolsonaro sentiment.

This figure depicts changes in participants' policy support towards abortion within the first three months of pregnancy (N = 5,618), zero deforestation (N = 5,632), social quotas (N = 3,610), and racial quotas (N = 3,587) among those who stated they would never vote for Lula or Bolsonaro in the first-round of the elections, and their difference in support (issue polarization), after versus before receiving misperception-correcting information about the respective policy. These effects were estimated using linear regression models controlling for age, sex, education, income and region, with clustered standard errors at the individual level. Data are presented as estimated mean changes, with error bars indicating 95% confidence intervals. All statistical tests were two-sided. The abortion and amazon treatments significantly reduced polarization over the respective policies. However, we did not find evidence that either the social quotas treatment or the racial quotas one did so (social quotas: b = 0.01, $t(1,682) = 0.14$, $p = 0.889$, 95% CI [-0.19, 0.22], standardized coefficient = 0.002; racial quotas: b = 0.12, $t(1,682) = 1.10$, $p = 0.272$, 95% CI [-0.10, 0.34], standardized coefficient = 0.001).

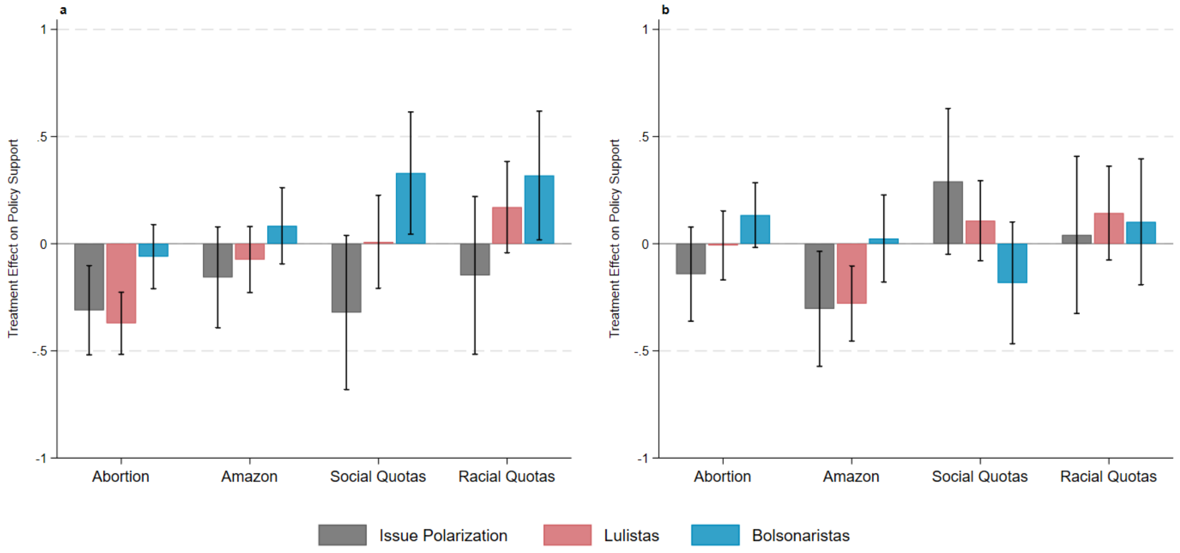

Supplementary Fig. 24 | The effect of providing misperception-correcting information on policy support by wave.

Panel a depicts changes in participants' policy support towards abortion within the first three months of pregnancy (N = 4,733) and zero deforestation (N = 4,744) in wave 2, and social quotas (N = 3,030), and racial quotas (N = 3,011) in wave 4, among respondents who reported intentions to vote for Lula (lulistas) or Bolsonaro (bolsonaristas) in the first election round, and their difference in support (issue polarization), after versus before receiving misperception-correcting information about the respective policy. Panel b depicts changes in participants' policy support towards abortion (N = 4,733) and zero deforestation (N = 4,744) in wave 3 and for social (N = 3,030) and racial (N = 3,011) quotas policies in wave 5. These effects were estimated using linear regression models controlling for age, sex, education, income and region, with clustered standard errors at the individual level. Data are presented as estimated mean changes, with error bars indicating 95% confidence intervals. All statistical tests were two-sided.

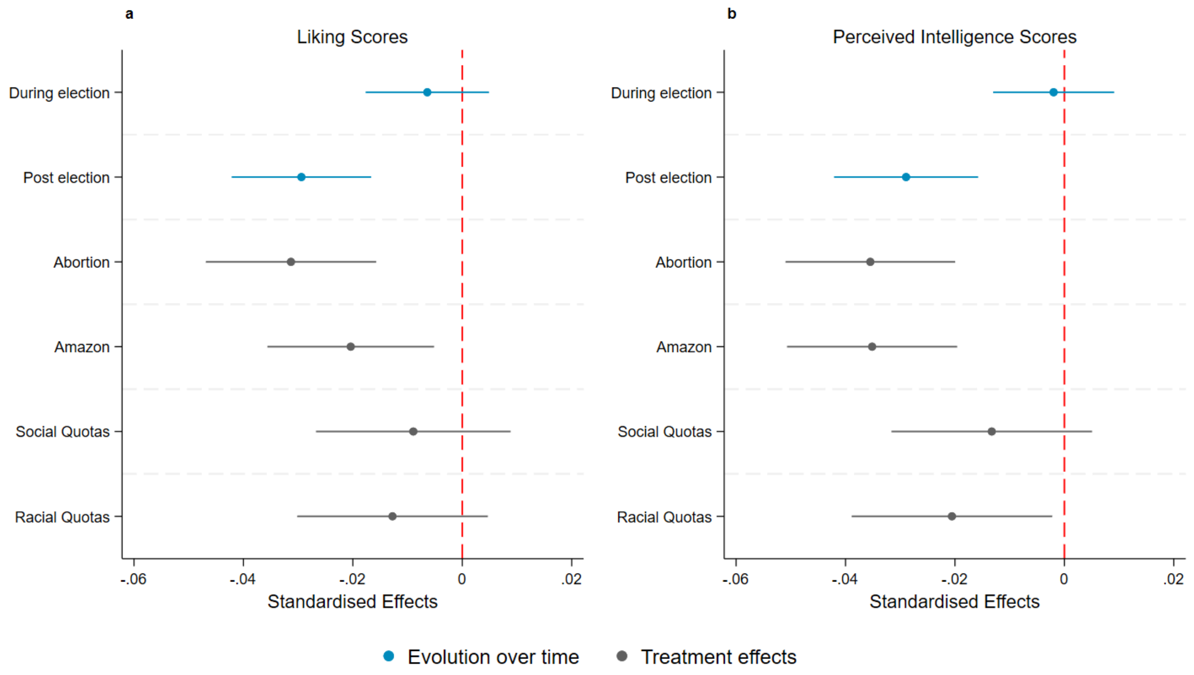

Supplementary Fig. 25 | Election and treatment effects

Panel a depicts the standardized change in average liking scores, compared to the pre-election, during and after the election period for political outgroups (blue coefficients; N = 13,594) and average standardized effects of misperception-correcting information across experimental conditions on liking scores for political outgroups (gray coefficients; N = 6,530). Panels b depicts equivalent effects for perceived intelligence. These effects were estimated using linear regression models with clustered standard errors at the individual level. Models for the election effects control for participants' age, sex, household income, education attainment, and region of residence, dummies for wave-specific treatment conditions. Error bars indicate 95% confidence intervals.

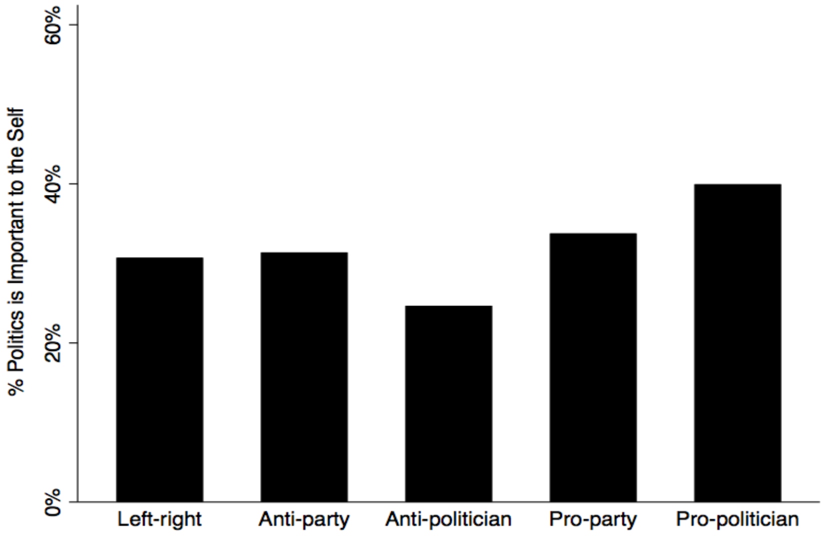

Supplementary Fig. 26 | Percentage of participants who reported that their political identity is one of the three most important aspects of their sense of identity in wave 3.

Political group definitions were randomized between respondents, allowing for comparison of the salience of the different group definitions.

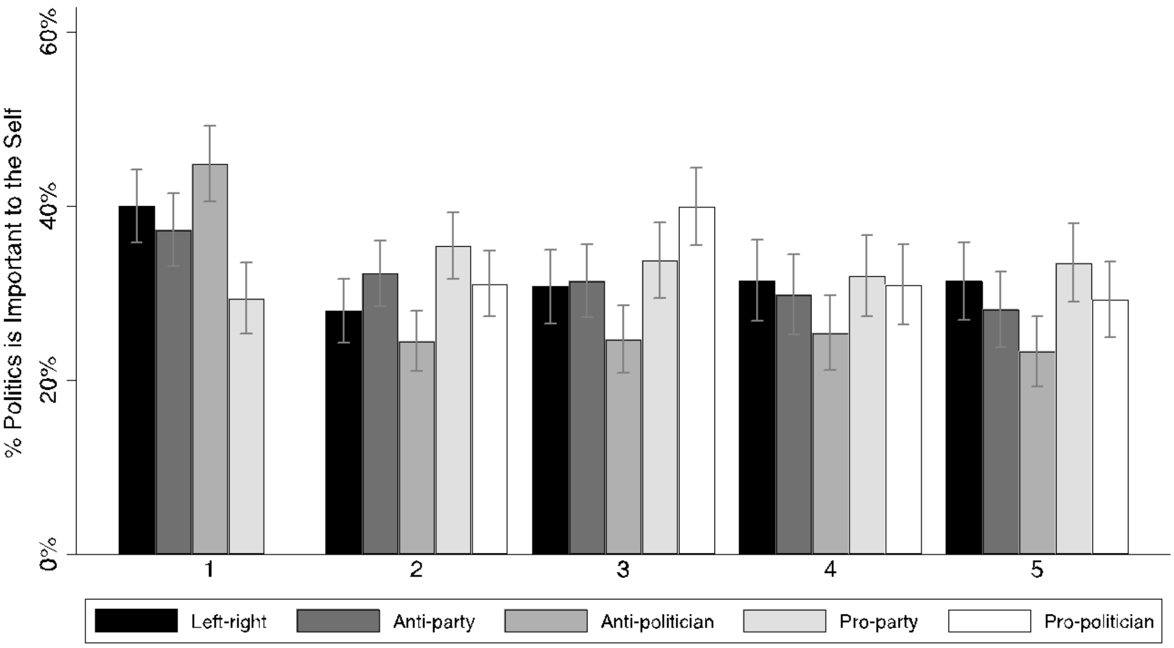

Supplementary Fig. 27 | Comparing the salience of political group definitions.

This figure shows the evolution of the percentage of respondents who reported that their political identity is one of the three most important aspects of their overall sense of identity across waves. Political group definitions were randomized between respondents within each wave, allowing for comparison of the salience of the different group definitions over time. Of note, the pro-politician grouping was the only political group definition to show a strong reduction in salience after the elections (b = -0.10, $t(1{,}798) = -3.71$, $p < 0.001$, 95% CI [-0.15, -0.05], standardized coefficient = -0.21).

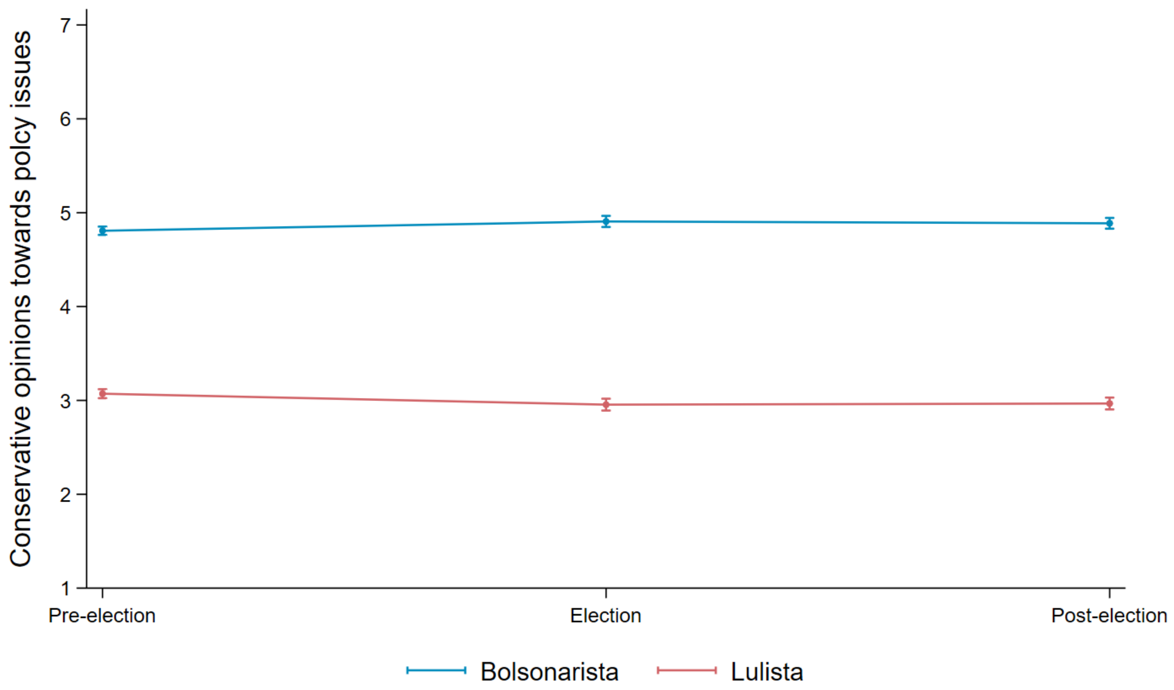

Supplementary Fig. 28 | Evolution of issue polarization for lulistas and bolsonaristas.

This figure depicts the evolving trends in issue polarization, defined as the difference between lulistas and bolsonaristas opinions towards policy issues, before, during, and after the election period (N = 14,006). The y-axis represents the average support for several controversial policies, including privatization, marijuana legalization, same-sex marriage (absent in wave 1), zero deforestation policies, gun ownership, severe penalties for corrupt public servants, sex education in schools, social quotas, racial quotas, promotion of christian values through public policies (absent in wave 1), wealth tax on fortunes (only in wave 1), and casino legalization (only in wave 1). Policy support was coded such that higher values reflect conservative opinions. The effects were estimated using a linear regression model. This figure considers as grouping criteria the reported intentions to vote for Lula (lulistas) or Bolsonaro (bolsonaristas) in the first election round. The model excludes participants who did not report intentions to vote for either Lula or Bolsonaro and were estimated with clustered standard errors at the individual level. Data are presented as model-predicted mean values, with error bars indicating 95% confidence intervals. All statistical tests were two-sided. Note: bolsonaristas held more conservative opinions than lulistas before the election (b = 1.74, $t(2,648) = 52.20$, $p < 0.001$, 95% CI [1.67, 1.80]). This polarization over policy issues further increased during (vs. before) the election (b = 0.22, $t(2,648) = 5.83$, $p < 0.001$, 95% CI [0.14, 0.29]) and remained steady after (vs. during) the election (b = 0.03, $t(2,648) = 0.96$, $p = 0.339$, 95% CI [-0.03, 0.10]).

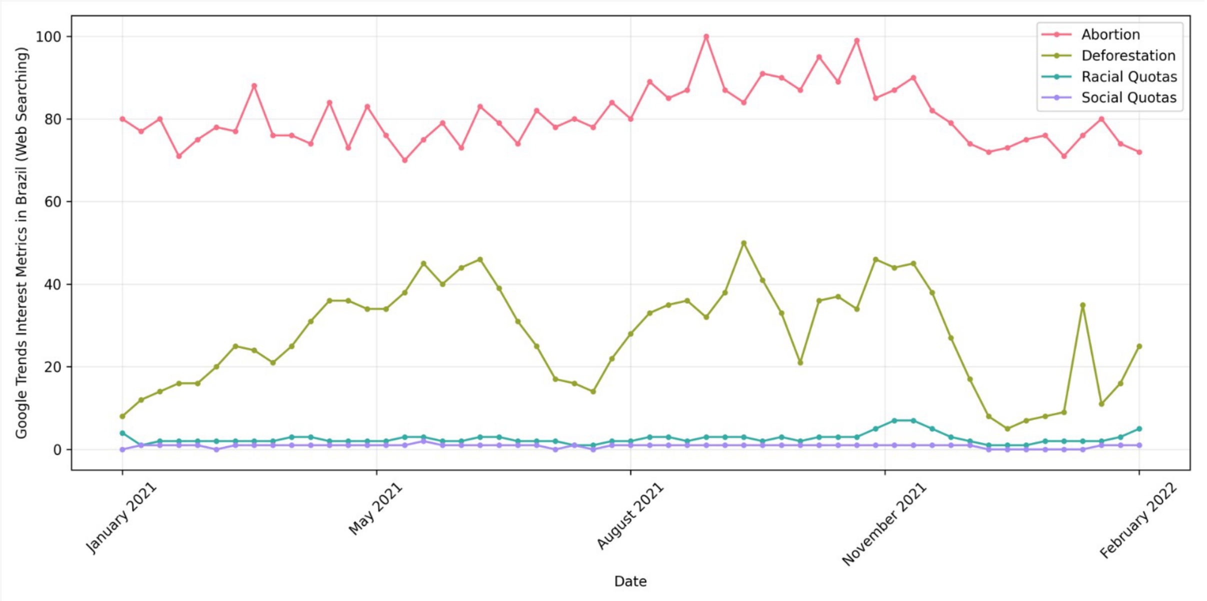

Supplementary Fig. 29 | Google trends on abortion, deforestation, racial and social quotas in Brazil from Jan 2021 to Feb 2022.

References

1. Johnston, C.D., Madson, G.J. Negativity bias, personality and political ideology. *Nat. Hum. Behav.* **6**, 666–676 (2022).

2. Makowski, D., Ben-Shachar, M., & Lüdecke, D. bayestestR: Describing Effects and their Uncertainty, Existence and Significance within the Bayesian Framework. *J. Open Source Softw.*, **4**(40) (2019).

3. Andraszewicz, S., Scheibehenne, B., Rieskamp, J., Grasman, R., Verhagen, J., & Wagenmakers, E.-J. An Introduction to Bayesian Hypothesis Testing for Management Research. *J. Manage.*, **41**(2), 521-543 (2015).

4. Lakens, D., Scheel, A. M., & Isager, P. M. Equivalence testing for psychological research: A tutorial. *Adv. Meth. Pract. Psychol. Sci.*, **1**(2), 259-269 (2018).

5. Voelkel, J. G. et al. Interventions reducing affective polarization do not necessarily improve anti-democratic attitudes. *Nat. Hum. Behav.* **7**, 55–64 (2022).
