# Supplementary material for: Dynamics of sociopolitical polarization and effects of misperception-correcting information around the 2022 Brazilian elections
Source: Nat Commun. 2026 Jun 5;17:4862. doi: 10.1038/s41467-026-72990-9 (PMC13241496; doi:10.1038/s41467-026-72990-9)
Supplement: Supplementary file 2 — Reporting Summary [file 41467_2026_72990_MOESM2_ESM.pdf]

nature portfolio

Corresponding author(s): Anna Petherick

Last updated by author(s): Sep 25, 2025

Reporting Summary

Nature Portfolio wishes to improve the reproducibility of the work that we publish. This form provides structure for consistency and transparency in reporting. For further information on Nature Portfolio policies, see our Editorial Policies and the Editorial Policy Checklist.

Statistics

For all statistical analyses, confirm that the following items are present in the figure legend, table legend, main text, or Methods section.

n/a	Confirmed	
☐	☒	The exact sample size (*n*) for each experimental group/condition, given as a discrete number and unit of measurement
☐	☒	A statement on whether measurements were taken from distinct samples or whether the same sample was measured repeatedly
☐	☒	The statistical test(s) used AND whether they are one- or two-sided<br>*Only common tests should be described solely by name; describe more complex techniques in the Methods section.*
☐	☒	A description of all covariates tested
☐	☒	A description of any assumptions or corrections, such as tests of normality and adjustment for multiple comparisons
☐	☒	A full description of the statistical parameters including central tendency (e.g. means) or other basic estimates (e.g. regression coefficient) AND variation (e.g. standard deviation) or associated estimates of uncertainty (e.g. confidence intervals)
☐	☒	For null hypothesis testing, the test statistic (e.g. *F*, *t*, *r*) with confidence intervals, effect sizes, degrees of freedom and *P* value noted<br>*Give P values as exact values whenever suitable.*
☐	☒	For Bayesian analysis, information on the choice of priors and Markov chain Monte Carlo settings
☐	☒	For hierarchical and complex designs, identification of the appropriate level for tests and full reporting of outcomes
☐	☒	Estimates of effect sizes (e.g. Cohen's *d*, Pearson's *r*), indicating how they were calculated

Our web collection on statistics for biologists contains articles on many of the points above.

Software and code

Policy information about availability of computer code

Data collection	Qualtrics
Data analysis	Stata 18; R Studio

For manuscripts utilizing custom algorithms or software that are central to the research but not yet described in published literature, software must be made available to editors and reviewers. We strongly encourage code deposition in a community repository (e.g. GitHub). See the Nature Portfolio guidelines for submitting code & software for further information.

Data

Policy information about availability of data

All manuscripts must include a data availability statement. This statement should provide the following information, where applicable:

- Accession codes, unique identifiers, or web links for publicly available datasets
- A description of any restrictions on data availability
- For clinical datasets or third party data, please ensure that the statement adheres to our policy

The data and analysis codes (Stata and R) that support the findings are available via: https://doi.org/10.17605/OSF.IO/Y8XKM

Research involving human participants, their data, or biological material

Policy information about studies with human participants or human data. See also policy information about sex, gender (identity/presentation), and sexual orientation and race, ethnicity and racism.

Reporting on sex and gender	"Sex", but not "gender" appears throughout the manuscript. The analysis for this study drew on a series of demographic survey items that act as controls, and that in the surveys were deliberately worded as per the Brazilian census. The item that we use to control for "sex"offers 3 response options male/female/prefer not to say, by self-categorization; here we added a 4th option that does not appear in the census: "other". The survey contains a number of questions specifically probing gender: masculinity, femininity, agreement (or otherwise) with traditional trait association of those concepts, and gender salience. While we have conducted exploratory associations delving into gender vs sex, we decided there was no space for these in this manuscript, and that the relevant arguments and literature was distinct (demanding potentially a separate paper).
Reporting on race, ethnicity, or other socially relevant groupings	Similarly, we draw on the Brazilian census for the survey item on race, and include a response option "don't know or prefer not to respond" (as additionally include a question on the salience of race for respondent sense of identity). As per gender, since this manuscript deals with theory that is brought together in a different literature. we do not include results subdivided by racial category, though this is of interest in the wider project.
Population characteristics	The study is representative of the Brazilian population. Geographical region, age, family income, education, in addition to race and gender, and political group identitification.
Recruitment	Participants were recruited online by survey company, Netquest, drawing on the company's panel. Approximately 2-3,000 respondents were recruited for each survey wave within quota categories by age, sex and household income.
Ethics oversight	Oxford University Ethics Committee (CUREC, Blavatnik School)

Note that full information on the approval of the study protocol must also be provided in the manuscript.

Field-specific reporting

Please select the one below that is the best fit for your research. If you are not sure, read the appropriate sections before making your selection.

☐ Life sciences ☒ Behavioural & social sciences ☐ Ecological, evolutionary & environmental sciences

For a reference copy of the document with all sections, see nature.com/documents/nr-reporting-summary-flat.pdf

Behavioural & social sciences study design

All studies must disclose on these points even when the disclosure is negative.

Study description	This study is a quantitative assessment of affective polarization, that uses responses to panel surveys with embedded experiments to assess both dynamic change and effects of different treatment conditions.
Research sample	The sample consists of approximately 2-3000 individuals, that are representative of the Brazilian population, drawn from a sampling frame compiled by Netquest of around 140,000 active members.
Sampling strategy	Netquest recruited the samples for each survey wave using within-region quotas by sex, age and household income.
Data collection	The survey waves were conducted via the internet without any direct interaction between participants and the researchers. Participants gave informed consent before completing surveys. Second-wave sample sizes were deliberately larger than necessary to achieve 80% power to detect small effect sizes, so as to allow for attrition across waves.
Timing	We collected data over five waves starting at (1) April 2022 (2) early July 2022; (3) October 2022; (4) late-November 2022; (5) late-January 2023.
Data exclusions	All waves included 3 attention checks and respondents that failed all were excluded from analysis.
Non-participation	Details of the panel survey design, including assignment of participants across each of the five survey waves can be found in Supplementary Information Table 3, and analysis of differential attrition in Supplementary Fig. 4.
Randomization	Respondents were randomly assigned to control, abortion or Amazon conditions in wave 2. Those assigned to abortion or Amazon in wave 2 received the alternative condition in wave 3. In wave 4, respondents were randomized independently of their assignments in prior waves, except that those assigned to control remained there across waves 4 and 5. Those assigned to social quota (vs racial quota) condition in wave 4 were counterbalanced in wave 5.

Reporting for specific materials, systems and methods

We require information from authors about some types of materials, experimental systems and methods used in many studies. Here, indicate whether each material, system or method listed is relevant to your study. If you are not sure if a list item applies to your research, read the appropriate section before selecting a response.

Materials & experimental systems

n/a	Involved in the study
☒	☐ Antibodies
☒	☐ Eukaryotic cell lines
☒	☐ Palaeontology and archaeology
☒	☐ Animals and other organisms
☒	☐ Clinical data
☒	☐ Dual use research of concern
☒	☐ Plants

Methods

n/a	Involved in the study
☒	☐ ChIP-seq
☒	☐ Flow cytometry
☒	☐ MRI-based neuroimaging

Plants

Seed stocks	*Report on the source of all seed stocks or other plant material used. If applicable, state the seed stock centre and catalogue number. If plant specimens were collected from the field, describe the collection location, date and sampling procedures.*
Novel plant genotypes	*Describe the methods by which all novel plant genotypes were produced. This includes those generated by transgenic approaches, gene editing, chemical/radiation-based mutagenesis and hybridization. For transgenic lines, describe the transformation method, the number of independent lines analyzed and the generation upon which experiments were performed. For gene-edited lines, describe the editor used, the endogenous sequence targeted for editing, the targeting guide RNA sequence (if applicable) and how the editor was applied.*
Authentication	*Describe any authentication procedures for each seed stock used or novel genotype generated. Describe any experiments used to assess the effect of a mutation and, where applicable, how potential secondary effects (e.g. second site T-DNA insertions, mosiacism, off-target gene editing) were examined.*
